# Fluorination: Simple Change but Complex Impact on Ferroelectric Nematic and Smectic Liquid Crystal Phases

**DOI:** 10.1021/jacs.4c16802

**Published:** 2025-02-07

**Authors:** Grant J. Strachan, Ewa Górecka, Jordan Hobbs, Damian Pociecha

**Affiliations:** † Faculty of Chemistry, University of Warsaw, ul. Pasteura 1, 02-093 Warsaw, Poland; ‡ School of Physics and Astronomy, University of Leeds, Leeds LS2 9JT, U.K.

## Abstract

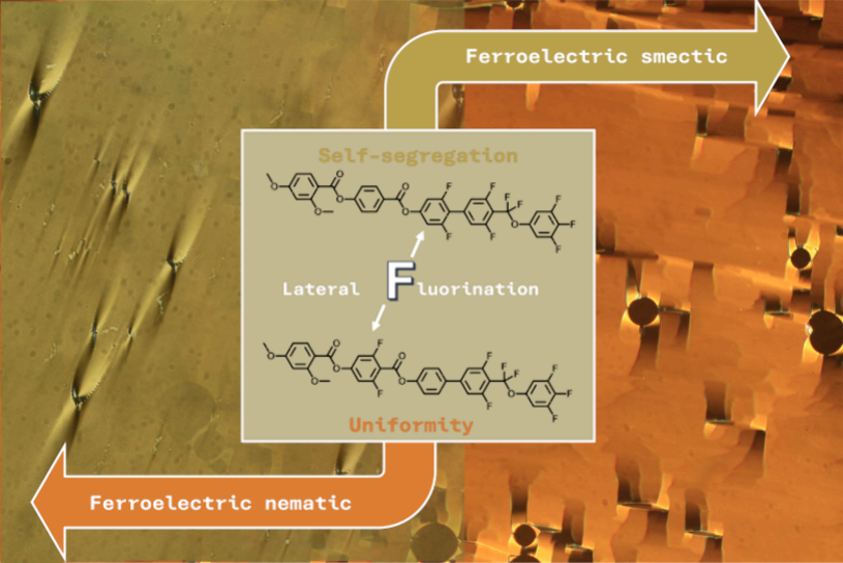

A series of liquid crystal (LC) materials are reported, which form a variety of ferroelectric nematic and smectic phases. The relationship between the number and position of lateral fluorine substituents and the formation of ferroelectric LC phases is investigated. While the addition of fluorine substituents increases the temperature at which ferroelectric order appears, the relationship between fluorination and the LC phase sequence is more complicated. Introducing lateral fluorine substituents can either suppress or promote the formation of ferroelectric smectic phases, depending on their position within the molecule, and the interplay between these trends allows for more exotic ferroelectric phases to appear.

## Introduction

**Figure 1:**
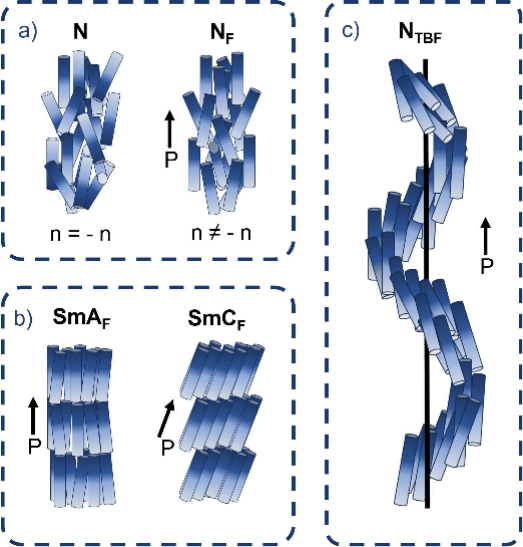
Schematic drawing of the structure of (a) paraelectric (N) and ferroelectric (N_F_) nematic phases, (b) orthogonal (SmA_F_) and tilted (SmC_F_) ferroelectric phases, and (c) heliconical ferroelectric nematic phase, N_TBF_. **P** is the electric polarization vector resulting from the parallel orientation of strongly dipolar molecules.

The recent discovery of the ferroelectric nematic (N_F_) phase^1−3^ has sparked a new era of research into fluid ferroelectric materials. The N_F_ phase is a nematic liquid crystal phase in which there is no positional order of molecules, but where they tend to align with their long axes pointing in the same average direction, known as the director, **n**. In the N_F_ phase, and in contrast to the conventional paraelectric nematic phase, N, inversion symmetry is lost such that **n ≠** −**n**, making the phase polar (Figure 1a).

While polar liquid crystal phases of chiral and bent-core materials have been studied for several decades, these were examples of improper ferroelectrics, and indeed, for many years, it was believed that thermal fluctuations in a liquid phase would prevent the observation of proper ferroelectricity. The discovery of the N_F_ phase proved this assumption incorrect, however, and now hundreds of ferroelectric nematogens have been reported, although in general, the majority of these are variations on three molecular archetypes, known as DIO,^2^ RM734,^1^ and UUQU-4N.^4^

In the past few years, the field of ferroelectric liquid crystals has been expanded by the reports of more complex ferroelectric LC phases, including orthogonal smectic A (SmA_F_) and tilted smectic C (SmC_F_) ferroelectric phases,^5−14^ as well as heliconical ferroelectric nematic (N_TBF_)^15^ and smectic C (SmC_P_^H^)^16^ phases (Figure 1). The ferroelectric SmA_F_ and SmC_F_ phases are characterized by lamellar order, as for conventional SmA and SmC phases, with the addition of polar order, and the direction of spontaneous electric polarization is along the director. In the helical phases (nematic and smectic), the director is tilted with respect to the helix axis, and the precession of the electric polarization around this axis means the polarization is partially compensated. However, these polar phases are still rare and not yet well-understood.

We have recently reported a fluorinated mesogenic compound which forms a sequence of ferroelectric nematic and smectic phases below the paraelectric nematic phase.^14^ This is a rare example of a mesogen forming an uninterrupted series of ferroelectric LC phases with increasing positional order. Another unusual feature of this material is the formation of two smectic phases, despite the molecule not containing any alkyl chains. Typically, the formation of conventional smectic LC phases is driven by self-segregation between mesogenic units and terminal chains, and this difference highlights the different relationships between the molecular structure and the formation of these new polar phases. We hypothesized that the formation of the ferroelectric smectic phases in this material is driven by a different type of molecular self-segregation, between fluorinated and nonfluorinated regions of the molecule, as the structure contains several lateral fluorine substituents, as do, to the best of our knowledge, all other examples of liquid crystals forming ferroelectric smectic or heliconical ferroelectric nematic phases.^5−14^ While this clearly indicates the importance of fluorine substituents in forming polar smectic phases, at first glance, there are no obvious trends linking the number and/or position of fluorine substituents and the phase behavior. The unique properties of fluorine substituents have played an important part in the development of many different liquid crystal phases;^17^ however, studies of these new polar phases have mainly focused on varying terminal chain length, the nature of linking groups, and the number of aromatic rings present, and there has not been a systematic study of the effects of fluorine substitution on the formation of these more ordered polar phases.

**Figure 2:**
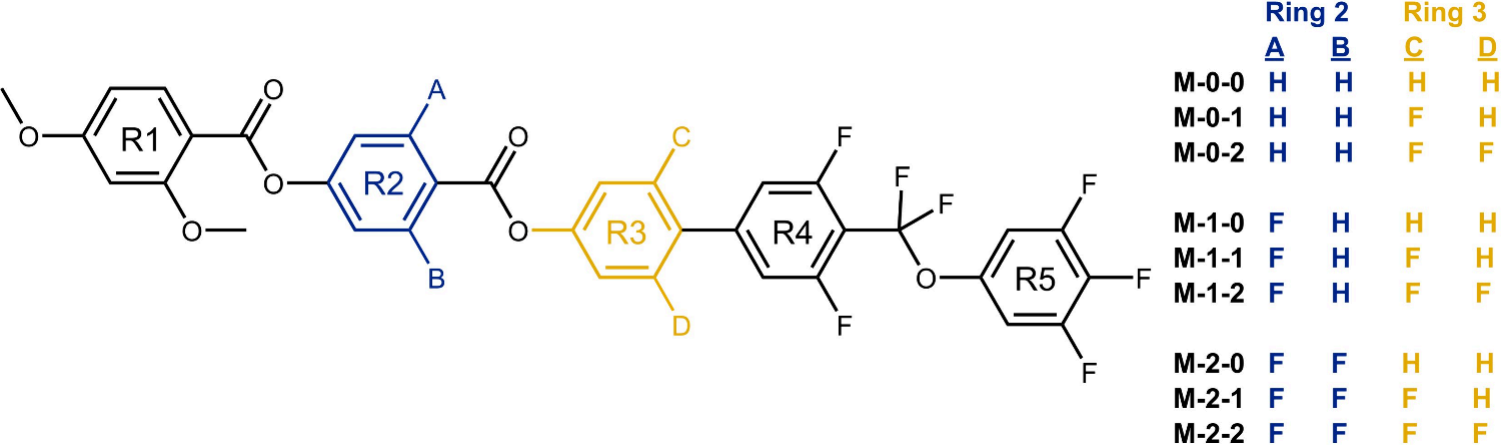
Structures of the materials reported here and their codes. **M-0-1** has been reported previously.^14^

In this study, we have investigated the effect of varying the number and position of lateral fluorine substituents on the formation of ferroelectric LC phases, and the structures of these materials are given in Figure 2. A description of the organic synthetic procedures and analytical characterization of the compounds is given in the Supporting Information. The materials have been given a code **M-*X*-*Y***, with *X* and *Y* representing the number of fluorine atoms (0, 1, or 2) on rings 2 and 3, respectively. The phase behavior of the new materials is given in Table 1.

**Table 1:** Transition Temperatures (in °C) and Phase Sequences for the Compounds in This Series

**M-**0-0	MP 147	SmC_F_	84^c^	SmA_F_	146	N_F_	152			N	267	Iso
**M-**0-1^d^	MP 141	SmC_F_	115	SmA_F_	167	N_F_	185			N	263	Iso
**M-**0-2	MP 138	SmC_F_	164^a^	SmA_F_	185	N_F_	220			N	252	Iso
**M-**1-0	MP 165	SmC_F_	72^b^	SmA_F_	134	N_F_	156^c^	N_X_	159^c^	N	262	Iso
**M-**1-1	MP 139	SmC_F_	108^b^	SmA_F_	130^a^	N_F_	191			N	253	Iso
**M-**1-2	MP 141	SmC_F_	143	N_TBF_	151^a^	N_F_	224			N	247	Iso
**M-**2-0	MP 169					N_F_	155^c^	N_X_	156^c^	N	238	Iso
**M-**2-1	MP 163					N_F_	185			N	233	Iso
**M-**2-2	MP 158					N_F_	214			N	231	Iso

a Taken from birefringence measurement.

b Taken from SAXS measurements.

c Determined from dielectric spectroscopy measurements.

d Taken from ref (14). MP stands for melting point.

## Results

### Assignment of LC Phases

**Figure 3:**
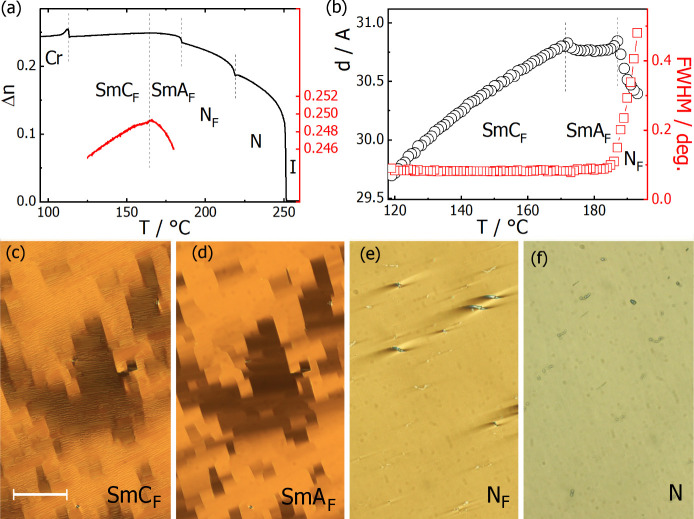
Compound **M-0-2**: (a) optical birefringence measured with green light (λ = 532 nm), red line representing enlarged dependence close to the SmA_F_–SmC_F_ phase transition (right axis), (b) layer spacing, *d*, and the diffraction signal width, fwhm, vs temperature, (c–f) optical textures of SmC_F_, SmA_F_, N_F_, and N phases taken in a 1.7-μm-thick cell treated for planar alignment, with parallel rubbing on both surfaces. Scale bar corresponds to 100 μm.

Preliminary assignment of LC phases was carried out using polarized light optical microscopy (Figure 3). The nematic phase in thin cells with planar anchoring and parallel rubbing on both surfaces produced a uniform texture. Identification of the N_X_ (also referred to as SmZ_A_^18^) phase was based on the observation of characteristic chevron defects (Figure S17a). The ferroelectric nematic, N_F_, phase produced a uniform texture in thin cells with characteristic conical defects anchored to glass pillars that serve as cell spacers.^19^ The N_TBF_ phase is characterized by a striped texture (Figure S17b), with the periodicity of the stripes reflecting the helical pitch.^15^ The SmA_F_ phase showed a uniform or a mosaic-like optical texture in thin cells with planar anchoring and parallel rubbing (Figure 3d). In the SmC_F_ phase, the mosaic texture usually breaks into small striped domains (Figure 3c); in other cases, a noncharacteristic and strongly scattering texture was observed.

X-ray diffraction measurements confirmed the assignment of the observed LC phases. In all of the nematic phases, only diffused signals were registered, showing a lack of positional order. On approaching the smectic phase, the low-angle diffraction signal narrows, and in the smectic phases, it reaches machine resolution (Figure 3b). The transition from orthogonal SmA_F_ to tilted SmC_F_ phase is marked by a decrease of layer thickness due to an increasing tilt angle (Figure S18).

The polar properties of the phases were confirmed by the observation of optical switching upon application of an electric field, which was accompanied by a switching current. The polarization calculated from the switching current peak is of the order of 4–5 μC cm^–2^, typical for proper ferroelectric liquid crystals, with no marked changes at the SmA_F_–SmC_F_ phase transition, showing the similar order of dipole moments in both smectic phases (Figure S19). Switching under a modified triangular-wave voltage (two subsequent voltage pulses of the same polarity are applied) clearly shows the ferroelectric nature of the ground state of the smectic phases. The first pulse induced a ferroelectric state that is preserved when the field is switched off, so the second pulse of the same polarity of electric field has no effect on the polar state of the sample and thus does not produce a switching current peak (Figure S19). In sufficiently high electric fields, in all polar phases, including the tilted SmC_F_ phase, the planar texture transforms to a homeotropic one, as the polarization is reoriented along the electric field (Figure S20). Moreover, in polar phases, a strong dielectric response at the low frequency of the applied field was detected by dielectric spectroscopy; such response served as an indication of the polar nature of the phase, although the exact interpretation of results of the dielectric spectroscopy data in strongly polar soft matter is still under debate.^20−22^

### Phase Behavior of Group I (M-0-*Y*)

**Figure 4:**
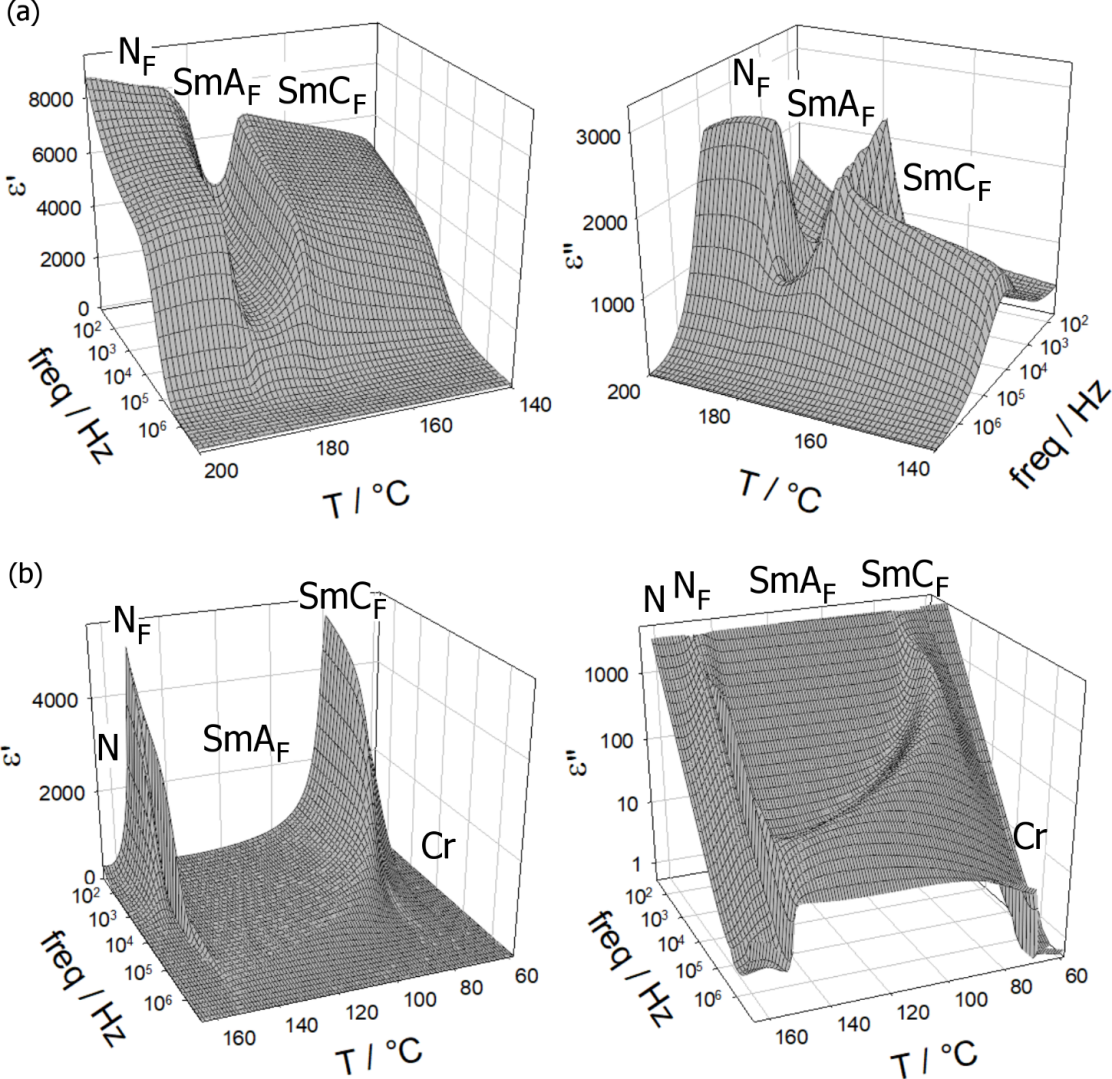
Real (ε′) and imaginary (ε″) parts of apparent dielectric permittivity of (a) **M-0-2** and (b) **M-0-0** compounds, measured in a 5-μm-thick cells with gold electrodes.

The materials with no fluorine substituents on ring 2 all formed a series of nematic and smectic liquid crystalline phases. All three **M-0-*Y*** materials exhibit N, N_F_, SmA_F_, and SmC_F_ phases. Measurement of the optical birefringence of **M-0-2** (Figure 3a) shows a continuous increase of Δn in the nematic phase, following a critical, power law dependence of the orientational order parameter (*S*) of the molecules. This trend continues into the N_F_ phase, although there is a pronounced dip at the N–N_F_ phase transition. Such a dip has been observed previously for other materials with the N–N_F_ phase sequence,^23^ and it indicates that the transition to the polar phase is accompanied by strong fluctuations in the orientational order, believed to be due to splay deformations. The N_F_–SmA_F_ transition is accompanied by a step-like jump in Δ*n*, which is typical for a weakly first-order transition and indicates that, similar to nonpolar systems, the formation of lamellar order is accompanied by a small increase in orientational order.^24^ In contrast, at the SmA_F_–SmC_F_ transition, a continuous decrease in Δn is seen, which may be ascribed to the distortion of the uniform texture by the formation of tilted domains. Such a temperature dependence of the optical birefringence seems to be general and was observed for all of the compounds studied here (Figure S21). The X-ray diffraction experiments performed for the smectic phases of **M-0-2** (Figure 3b) show that the layer spacing is essentially constant in the SmA_F_ phase and corresponds to the length of the molecule, while it continuously decreases on cooling through the SmC_F_ phase, consistent with the assumption that molecules tilt in the layers. Dielectric spectroscopy studies of **M-0-2** are consistent with the polar phase assignments (Figure 4a). They revealed a strong dielectric response in the N_F_ and SmC_F_ phases due to the strong fluctuations related to the easy reorientation of the polarization vector. In the SmA_F_ phase, the response is weaker, as any fluctuation of the polarization vector in this phase is coupled to the smectic layer reorientation and thus is energetically costly.

This behavior observed for **M-0-2** is essentially identical to that previously reported for **M-0-1**,^14^ although the transition temperatures of the polar phases of **M-0-2** are markedly higher than those of **M-0-1**. The behavior of **M-0-0** is also very similar; however, both *T*_NF-N_ and *T*_NF-SmAF_ are lower, and the temperature range of the SmA_F_ phase is considerably increased in comparison with **M-0-2**. The broad temperature range of the orthogonal SmA_F_ phase allowed the development of polarization fluctuations to be followed on approaching the tilted smectic phase. On cooling, the relaxation frequency of the mode decreases, while its strength increases, and it appears that this soft mode is related to instantaneous tilt fluctuations (Figure 4b).

### Phase Behavior of Group II (M-1-*Y*)

**Figure 5:**
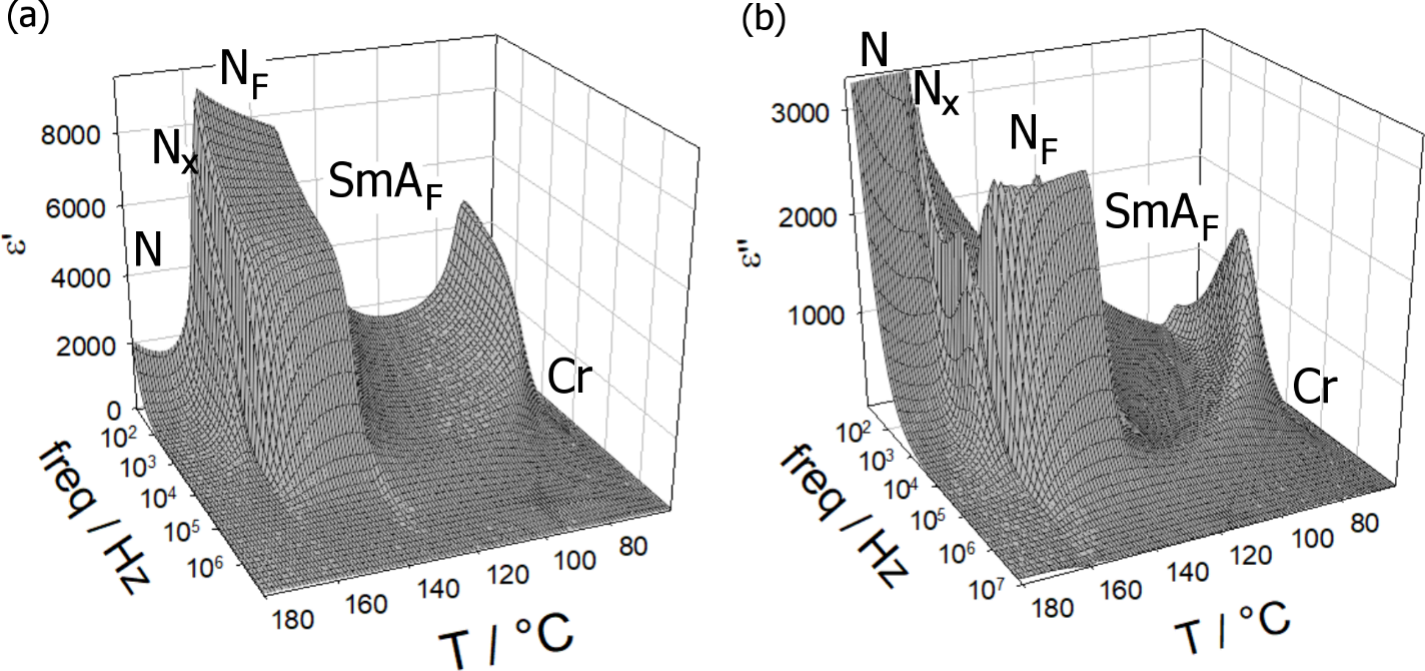
Real (a) and imaginary (b) parts of the apparent dielectric permittivity of **M-1-0** measured in a 5-μm-thick cell with gold electrodes.

The next group of materials has one fluorine substituent on ring 2. The first two of these, **M-1-0** and **M-1-1**, show very similar phase behavior to their analogues in group I, **M-0-0** and **M-0-1**, respectively. In addition to forming N, N_F,_ SmA_F_, and SmC_F_ phases, **M-1-0** also forms an N_X_ phase in a narrow temperature range, and the phase could be easily identified in microscopic studies by the formation of characteristic chevron defects (Figure S17). Dielectric spectroscopy studies for this compound (Figure 5) show a mode beginning to develop in the nematic phase, which rapidly decreases in intensity at the transition to the N_X_ phase, before strongly increasing on entering the N_F_ phase. The behavior across the N_F_ and SmA_F_ phases matches that previously described for materials in group **M-0-*Y***. On cooling through the SmA_F_ phase, a mode related to tilt fluctuations begins to develop; however, **M-1-0** often easily crystallizes before reaching the SmC_F_ phase. **M-1-1** shows the same phase sequence as the analogous **M-0-1**, with similar transition temperatures, except for a pronounced decrease in the N_F_–SmA_F_ transition temperature, which is over 30 K lower for **M-1-1**.

**Figure 6:**
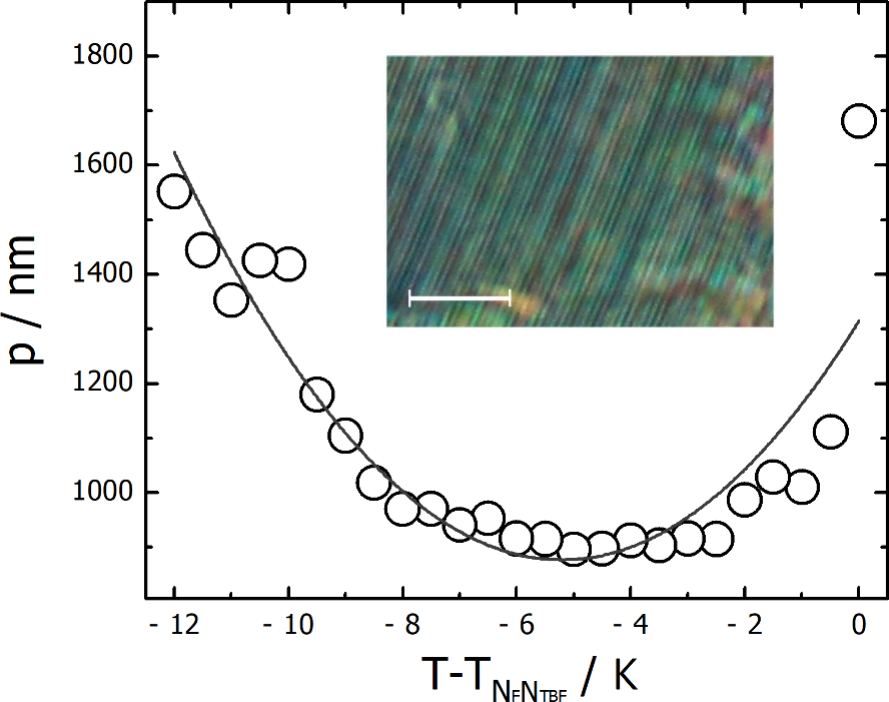
Temperature dependence of the helical pitch (circles) in the N_TBF_ phase of **M-1-2** on cooling measured by diffraction of laser light from a one-surface-free sample; line is a guide-for-eye. In the inset, optical texture of the one-surface-free sample prepared on a glass plate without surface treatment. The stripes are clearly visible which give rise to light diffraction. Scale bar corresponds to 20 μm.

The final compound in this group, **M-1-2**, also forms paraelectric and ferroelectric nematic phases, but uniquely among the materials discussed here, it also forms a heliconical ferroelectric nematic phase, N_TBF_, in which the molecules become tilted and make the precession on the tilt cone, forming a helix. The properties of this phase were very similar to that reported for the first observed N_TBF_ material.^15^ The formation of the heliconical structure of the N_TBF_ phase causes a decrease in the optical birefringence (Figure S21), as the inclination of molecules from the helix axis lowers the extraordinary refractive index and simultaneously increases the ordinary one. Formation of the helix, which has a pitch of the order of micron, is responsible for striped optical textures (Figure S17) and also gives rise to diffraction patterns observed when the sample is illuminated with laser light. The pitch length, measured in light diffraction experiments with one-surface-free or film samples, increases on approaching the transition to the N_F_ phase but also to the smectic C_F_ phase, suggesting that the helix unwinds at both transitions (Figure 6). In thin cells, where the influence of surfaces is strong, the helix was less temperature-dependent. Whether the SmC_F_ phase has a helical structure is ambiguous, as strong optical scattering prevents clear diffraction measurements. Since the helix in the N_TBF_ phase tends to unwind on approaching the transition to the smectic phase, it is reasonable to assume that the phase is nonhelical. A similar unwinding of the helix was reported in the ^HC^NF phase reported by Nishikawa et al., approaching the transition to a nonhelical smectic phase.^11^ The dielectric spectroscopy measurements of **M-1-2** show some decrease of permittivity in the N_TBF_ phase compared to the N_F_ phase (Figure S22).

### Phase Behavior of Group III (M-2-*Y*)

**Figure 7:**
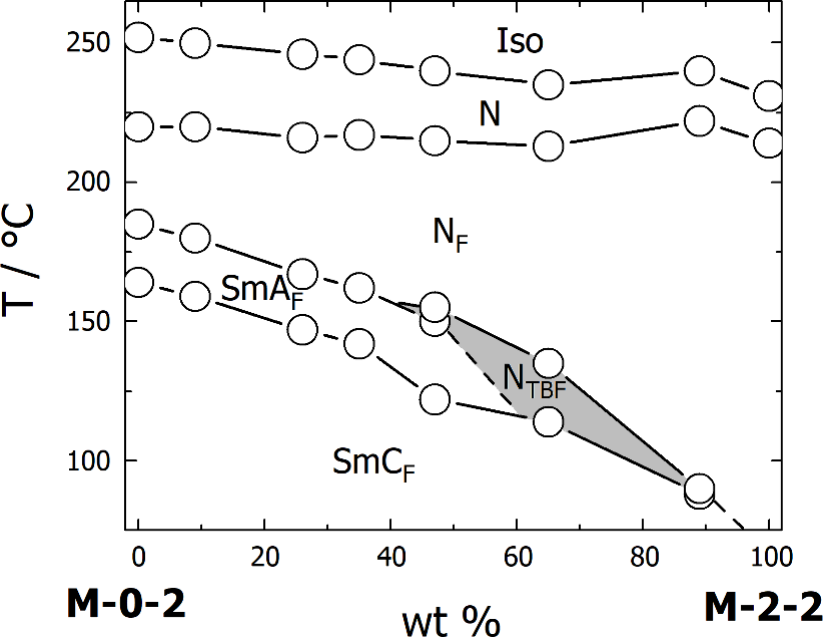
Phase diagram for binary mixtures of **M-0-2** and **M-2-2**.

The materials of group III, **M-2-Y**, show a more limited range of liquid crystalline phases. All three form N and N_F_ phases, while **M-2-0** also forms an N_X_ phase, as seen in the analogous material **M-1-0**. Dielectric spectroscopy revealed a strong relaxation mode in the temperature range of the N_F_ phase (Figure S23). Although the N–N_F_ phase transition temperatures for these compounds are comparable to those of analogous compounds of groups I and II, no additional LC phases were seen below the N_F_ phase. While these materials did have a higher melting point than many of the others reported here, they could be supercooled well below this, in some cases reaching below 70 °C, so the lack of smectic or other LC phase behavior does not appear to be simply due to crystallization preventing their observation. In order to estimate the possible onset of smectic behavior in **M-2-2**, a binary mixture with **M-0-2** (the material with the widest range of smectic phases) was studied. By the extrapolation of the trends visible in the constructed phase diagram (Figure 7), it can be estimated that the smectic phase in **M-2-2** should be formed below 70 °C. Interestingly, for mixtures with a concentration of **M-2-2** above 50%, the phase sequence changes, and N_TBF_ was observed, which did not appear in either of the two pure components.

## Discussion

### Trends in Phase Sequences and Transition Temperatures

**Figure 8:**
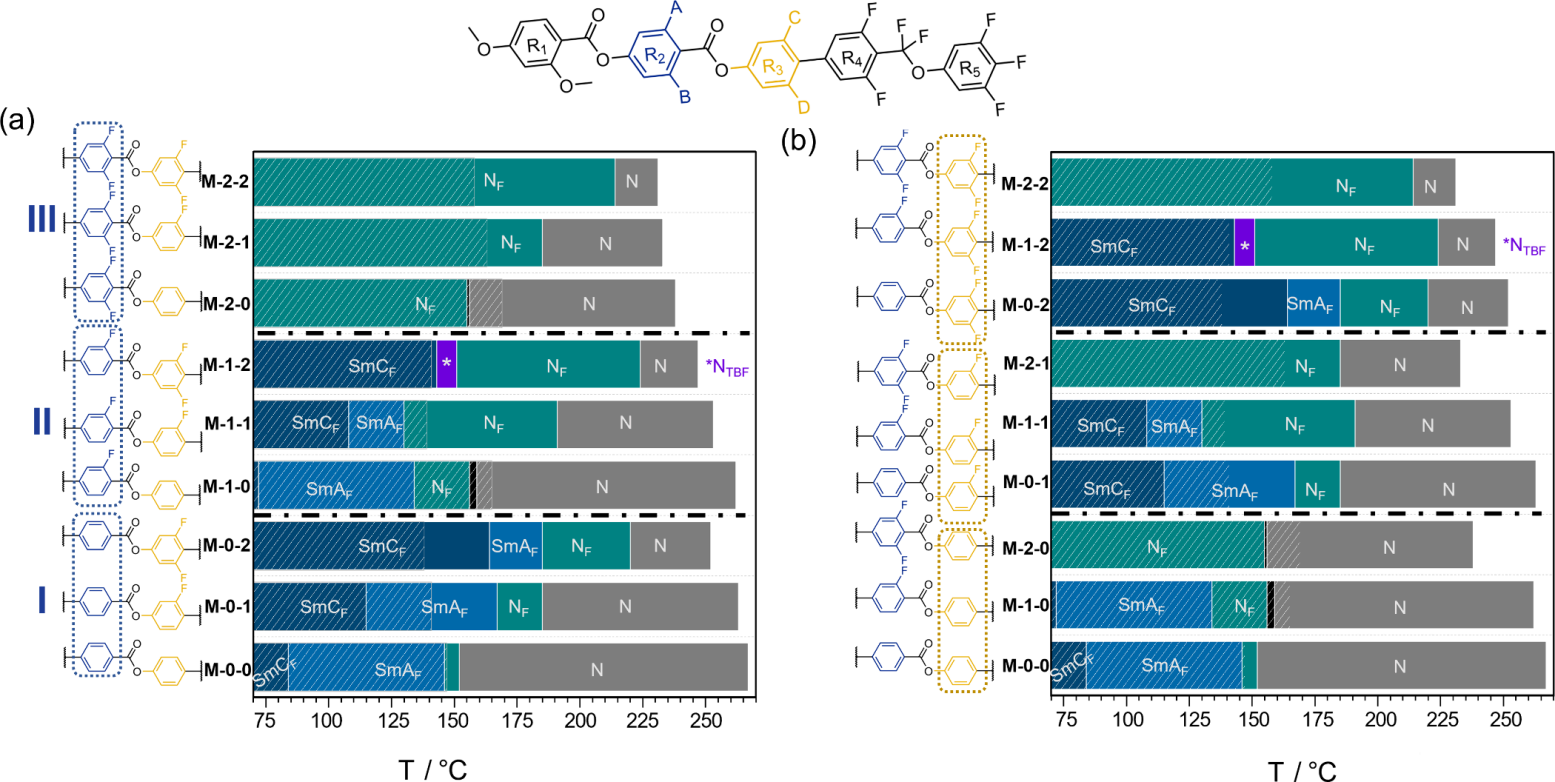
Phase sequences formed by the nine compounds reported. They are ordered into groups by (a) number of fluorine atoms on ring 2 or (b) number of fluorine atoms on ring 3. The black bars represent the N_X_ phase, and white hashed lines indicate the melting point.

The simplest factor to consider when comparing the LC properties of materials is molecular shape anisotropy, which here is primarily affected by the number of fluorine substituents in the molecular core. However, it is apparent that such a simplification does not represent the complex relationship between molecular structure and LC phase formation present in the series of mesogens studied here. In addition, the typical structural feature driving the formation of smectic phases is the nanosegregation between different parts of the molecule, particularly between alkyl chains and aromatic cores. However, this also does not apply to the molecules studied here that have no terminal or lateral alkyl chains. It is clear, therefore, that other factors must be at play in these materials. To investigate the possibilities, we will first consider the effects of adding fluorine substituents on ring 3 for materials with the same substitution pattern on ring 2 (Figure 8a).

For groups I and II, increasing the number of fluorine substituents leads to a decrease of the clearing point, reflecting the lowering of molecular shape anisotropy. On the other hand, the addition of fluorine atoms strongly increases the temperature at which the ferroelectric nematic phase appears, by ∼30 K per additional fluorine atom, which can be ascribed to the increase in the molecular dipole moment (Table 2). For the compounds of group I, having no fluorine atoms on ring 2 (**M-0-0**, **M-0-1**, and **M-0-2**), there is also a clear trend regarding SmA_F_–N_F_ and SmC_F_–SmA_F_ phase transition temperatures, which increase considerably on additional fluorination. For the materials of group II, having one fluorine substituent on ring 2, the effect of the addition of fluorine substituents on ring 3 is more complicated. Unlike in group I, different sequences of LC phases are observed for the three materials: **M-1-0**, **M-1-1**, and **M-1-2**. The mesogen **M-1-0** forms an additional N_X_ phase between the N and N_F_ phases, although it has a very short temperature range. The difference in smectic behavior is more significant. The addition of one fluorine atom (**M-1-1**) actually decreases the SmA_F_–N_F_ transition temperature by 4 K, while in **M-1-2**, with two fluorine atoms on ring 3, the SmA_F_ phase is not observed, and instead, an N_TBF_ phase is formed. The temperature of the transition to the SmC_F_ phase is increased by the addition of fluorine substituents on ring 3, as was observed for group I, although these are lower than those for the corresponding materials in group I.

**Table 2:** Molecular Dipole Moments Calculated at the B3LYP-GD3BJ/cc-pVTZ Level of DFT

**M-**0-0	12.30 D
**M-**0-1	13.29 D
**M-**0-2	13.71 D
**M-**1-0	13.00 D
**M-**1-1	13.99 D
**M-**1-2	14.48 D
**M-**2-0	13.35 D
**M-**2-1	14.38 D
**M-**2-2	14.80 D

Next to consider is the effect of adding fluorine substituents on ring 2, which can be seen by comparing materials with the same substituents on ring 3, i.e., the three sets **M-*X*-0**, **M-*X*-1**, and **M-*X*-2** (Figure 8b).

In all three sets, adding one fluorine on ring 2 decreases *T*_NI_ only slightly, while the addition of a second fluorine atom leads to a decrease of ca. 20 K. The N_F_–N transition temperature is much less sensitive to fluorination on ring 2 than on ring 3, with the addition of the first fluorine substituent leading to an increase of only around 5 K, while the addition of a second fluorine atom actually leads to a decrease of ca. 10 K compared to the analogous materials with a single fluorine substituent on ring 2. The formation of an antiferroelectric nematic, N_X_, phase is observed for compounds **M-1-0** and **M-2-0**, and it appears that the formation of this phase requires at least one fluorine substituent on ring 2 and no fluorine substituents on ring 3. The addition of fluorine substituents on ring 2 appears to suppress the formation of ferroelectric smectic phases, with the addition of one fluorine substituent decreasing both the SmA_F_–N_F_ and SmC_F_–SmA_F_ transition temperatures, and the addition of a second fluorine leading to a loss of all smectic behavior. The negative effect caused by fluorination of ring 2 on the formation of the SmA_F_ phase appears to be strengthened by the presence of fluorine substituents on ring 3. Comparing **M-0-0** and **M-1-0**, the SmA_F_–N_F_ transition of the latter is 12 K lower; for **M-0-1** and **M-1-1**, it is 37 K lower; while in **M-1-2**, the SmA_F_ phase is lost entirely, and the N_TBF_ phase is revealed.

To summarize these observations, it is apparent that the addition of fluorine substituents on ring 3 has relatively little effect on the N-Iso transition, while increasing the temperature onset of polar order by ca. 30 K per fluorine substituent. Fluorination of ring 2 has a much smaller effect on the N_F_–N transition but appears to suppress the tendency for smectic ordering.

### Relationships between Molecular Structure and Liquid Crystallinity

Since the first reports of the N_F_ phase, a great deal of importance has been placed on the need for large molecular dipole moments.^25^ While more recently reported materials have shown that this is not the sole feature driving the formation of ferroelectric LC phases, it is still an important factor. The dipole moments of molecules studied here were calculated using DFT at the B3LYP-GD3BJ/cc-pVTZ level, and the results are given in Table 2. All the materials have strong dipole moments of a similar magnitude to those reported for other molecules forming N_F_, N_TBF_, and ferroelectric smectic phases. In general, the addition of a fluorine substituent increases the strength of the molecular dipole, but the scale of this increase depends on the position of the F atom in the mesogenic core and the overall number of fluorine substituents per molecule. The addition of the first fluorine atom to ring 3 leads to an increase in the dipole moment of ca. 1 D, while the second fluorine atom only produces an increase of ca. 0.5 D. Moreover, when the fluorine atoms are added to ring 2, the corresponding increase in dipole moment is smaller, ca. 0.7 and 0.4 D, for the first and second substituents, respectively. This difference has also been reported in dipole moments calculated for other ferronematogens.^26^ However, it is clear that the change of the dipole moment is not solely responsible for different phases formed by these materials; one should also consider changes in the distribution of electron density within the molecule caused by changing the number and position of the lateral fluorine substituents.

**Figure 9:**
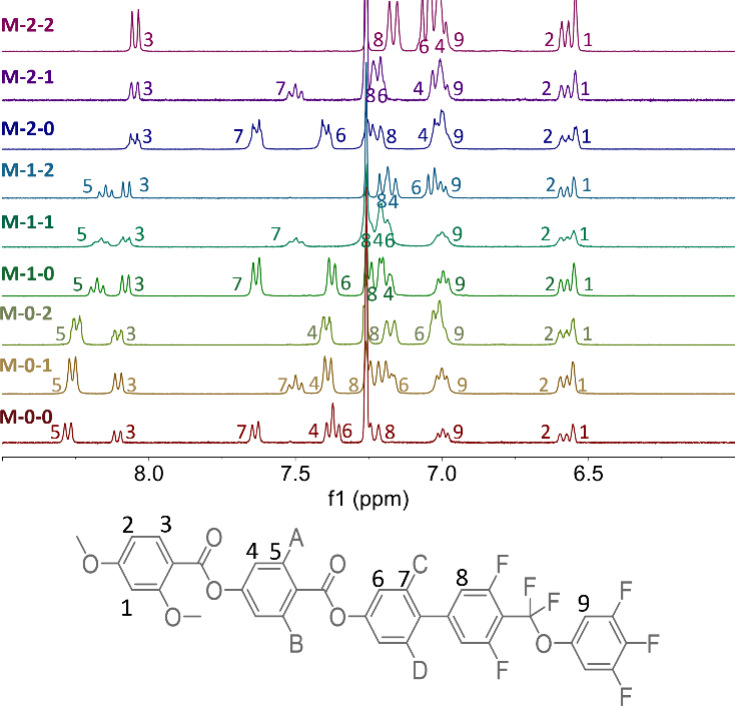
Aromatic region of the ^1^H solution phase NMR spectra measured in CDCl_3_ of the materials reported here.

Perhaps the simplest approach to gain some experimental insight into electron density distribution is to compare the changes seen in the ^1^H NMR spectra, (Figure 9), as the chemical shift (δ) of the protons in a molecule depends on the electron distribution. The largest changes in shift are seen for protons on the ring to which a fluorine substituent is added, as expected for the addition of a nearby, highly electronegative atom (Table 3). The effects of introducing fluorine substituents are not localized to an individual aromatic ring, although the effect on a neighboring ring is an order of magnitude smaller. When a fluorine atom is added to ring 3, the chemical shift of protons on ring 2 is also affected, suggesting that there is an increase in electron density at position 5 and a decrease at position 4. In addition, H_8_ is also affected when two fluorine substituents are added to ring 3, although this presumably includes through-space effects due to the shorter separation of the rings in a biphenyl group, in addition to changes of the electronic distribution within the molecule. In contrast, when a fluorine atom is added to ring 2, protons on rings 1 and 3 are affected. H_6_ experiences a deshielding effect, indicating a decrease in the surrounding electron density, while in contrast, H_3_ is shielded. In combination, these observations show that, as well as the large local increase in electron density at the position of the fluorine substituent, more subtle changes in the electron distribution are seen across the whole molecule.

**Table 3:** Change in the ^1^H Chemical Shift after Addition of Fluorine Substituents^a^

		Δδ/ppm			
ring	proton	+1F ring 2	+2F ring 2	+1F ring 3	+2F ring 3
1	**H**_**3**_	–0.02	–0.05	0	0
2	**H**_**4**_	–0.2	–0.4	0.01	0.02
2	**H**_**5**_	–0.1		–0.01	–0.03
3	**H**_**6**_	0.01	0.03	–0.2	–0.36
3	**H**_**7**_	0	0	–0.14	
4	**H**_**8**_	0	0	0	–0.04

a Values given are an average of the shift difference for the three sets of molecules and were measured in a dilute CDCl_3_ solution. Negative values correspond to shielding of the ^1^H nuclei.

To better visualize the electron distribution in these molecules, the electrostatic potential energy surfaces were calculated for the DFT-optimized geometries. In order to compare the effect of structural modifications, one-dimensional electrostatic potential (ESP) profiles were generated by radially averaging the electrostatic potential along the *Z*-axis (long axis) of the molecules for a fixed value of the electron density, according to previously reported methods.^10,27^

**Figure 10:**
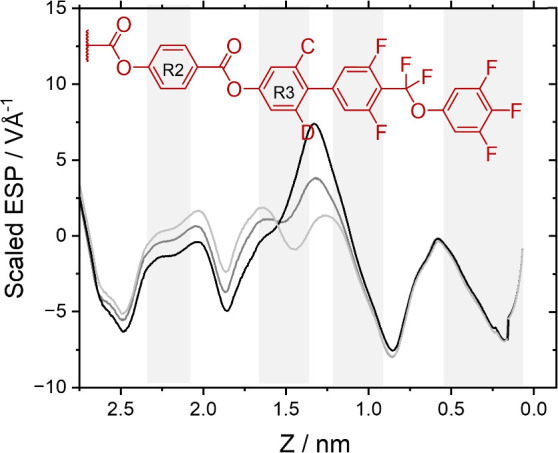
1D representations of the electrostatic potential along the long axis of the molecules in group I: **M-0-0** (black), **M-0-1** (dark gray), and **M-0-2** (light gray). The methoxyphenyl ring 1 has been omitted for clarity.

The addition of fluorine substituents has a clear effect on the electrostatic potential, with the greatest difference seen close to the position of the substituents, as would be expected. Considering first the effect of adding fluorine to ring 3, the same trends are seen in all three groups. Examining the 1D ESP profile for **M-0-0**, a series of alternating minima and maxima are seen (Figure 10), with the deepest minima (the regions of greatest electron density) corresponding to the fluorinated rings 4 and 5, with contributions from the fluoroether link. Two additional minima are seen, corresponding to the ester groups. A maximum in the ESP is centered around the biphenyl unit. Adding a fluorine substituent to ring 3 (**M-0-1**) decreases the height of this maximum and introduces a slight shoulder while the addition of the second fluorine atom (**M-0-2**) produces a distinct, though small, minimum and leads to an almost sinusoidal variation in the ESP across the biphenyl unit. In addition, the presence of fluorine substituents on ring 3 shifts the ESP around ring 2 to more positive values. These trends are also seen for the materials with 1 and 2 fluorine substituents on ring 2 (Figure S24).

**Figure 11:**
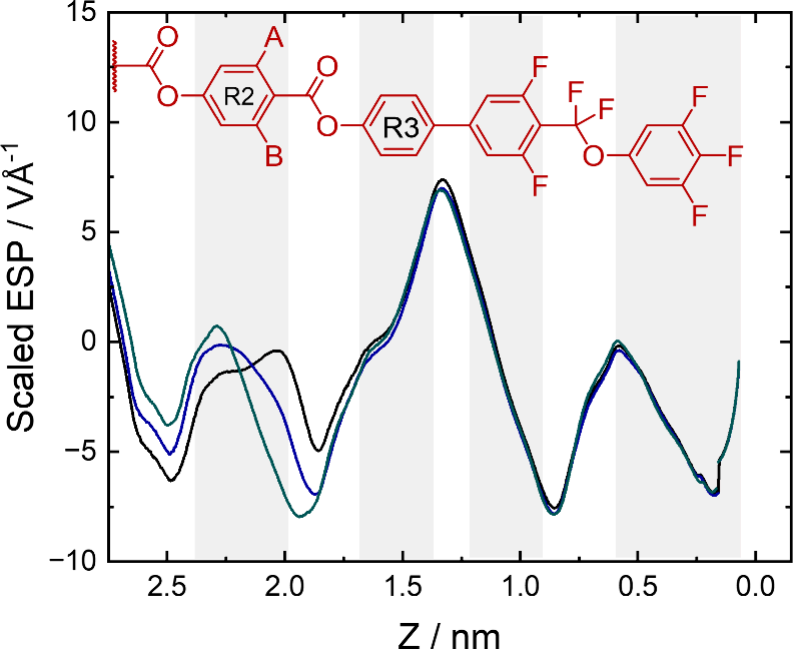
1D representations of the electrostatic potential along the long axis of the molecules with no fluorine substituents on ring 3: **M-0-0** (black), **M-1-0** (blue), and **M-2-0** (green). The methoxyphenyl ring 1 is omitted for clarity.

Next, we can consider the effect of adding fluorine substituents to ring 2 (Figure 11). This appears to have a more localized effect on the ESP, with no notable changes seen in the regions of rings 3, 4, or 5. It appears that the additional electron density introduced by the fluorine atom “combines” with the ester group linking rings 2 and 3, leading to an increase in the depth and breadth of the corresponding minimum in the ESP, and resulting in a smoother variation in the ESP profile between the two ester groups.

Combining these observations with the trends seen in the LC behavior of these materials, a few patterns can be noticed. First, materials with two fluorine substituents on ring 3 have a smoother variation of ESP and similar heights of the maxima (across rings 2–5) (Figure S24f) and have the highest onset temperatures of polar LC phases (**M-0-2**, **M-1-2**, and **M-2-2**). Second, the materials with two fluorine substituents on ring 2 (**M-2-0**, **M-2-1**, and **M-2-2**) do not form any smectic phases. Compared to the other materials, these have the largest ESP minima around the ester group linking rings 2 and 3, and it is of a comparable strength to the minima centered around the fluoroether group (Figure S24c). This may indicate that, while alternating regions of positive and negative charge are required for the formation of polar phases, differences in the relative intensities of charge densities at different ends of the molecule could help drive the formation of polar smectic phases. The antiferroelectric nematic (N_X_) phase was only seen for **M-1-0** and **M-2-0**, both of which have a clear maximum in the ESP near the center of the molecule, (Figure S24d), which may indicate that this interferes somewhat with the parallel alignment of molecules required to form the N_F_ phase, allowing an intermediate N_X_ phase to be observed below the paraelectric nematic phase.

The electron density distribution is expected to be most uneven for compound **M-0-2**, and indeed it shows the broadest range of smectic phases, while the most even electron density distribution seen for **M-2-2** seems to be responsible for the suppression of smectic behavior and the formation of only nematic phases. In an intermediate case, for compound **M-1-2**, the helical N_TBF_ phase is found, and this behavior is reproduced also for the mixtures of **M-2-2** and **M-0-2**: an island of N_TBF_ phase is observed between N_F_ and smectic phases in the middle of the binary phase diagram.

## Conclusions

We have synthesized a series of polar liquid crystals to investigate the influence of lateral fluorination on the formation of ferroelectric LC phases. In general, the addition of fluorine substituents increases the temperature at which ferroelectric phases appear, but the sequence of the LC phases formed is highly sensitive to both the number and position of the fluorine substituents. For materials **M-0-0**, **M-0-1**, and **M-0-2**, the addition of fluorine substituents on ring 3 (part of the biphenyl unit) promotes the formation of ferroelectric smectic phases. In contrast, the addition of the F atom to ring 2 (between the ester links) in **M-0-0**, **M-1-0**, and **M-2-0** has the opposite effect. The appearance of smectic phases in molecules lacking terminal chains is somewhat surprising, as the self-segregation of alkyl terminal groups and aromatic cores is typically the primary mechanism behind layer formation. We speculate that, for the studied materials, strong interactions between specific molecular fragments with differing electron densities may serve as an alternative mechanism facilitating the formation of layers. Such a mechanism is expected to be stronger upon the addition of fluorine substituents on ring 3 than on ring 2, as this increases the discrepancy between the magnitude of the variation of electron density at the two ends of the molecule, that is, the regions of increased electron density toward the fluoroether region are more pronounced than those at the methoxyphenyl end of the molecule. This drives the formation of polar smectic phases. In contrast, fluorination on ring 2 leads to a more uniform variation in electron density along the length of the molecule and favors the formation of the N_F_ phase. Unexpectedly, the interplay between competing effects allows for the observation of spontaneously heliconical polar nematic, the N_TBF_ phase in **M-1-2**, with fluorine substituents on both rings 2 and 3. These results highlight the crucial importance of molecular structure for the formation of polar liquid crystals and the highly sensitive relationship between fluorination patterns and the type of LC phase produced.

## Supporting Information Available

Full experimental details, synthetic procedures and characterization details for organic compounds, and additional characterization data for liquid crystal phases (PDF)

## References

[ref1] Mandle R. J.; Cowling S. J.; Goodby J. W. A Nematic to Nematic Transformation Exhibited by a Rod-like Liquid Crystal. Phys. Chem. Chem. Phys. 2017, 19 (18), 11429–11435. 10.1039/C7CP00456G. 28422219

[ref2] Nishikawa H.; Shiroshita K.; Higuchi H.; Okumura Y.; Haseba Y.; Yamamoto S.; Sago K.; Kikuchi H. A Fluid Liquid-Crystal Material with Highly Polar Order. Adv. Mater. 2017, 29 (43), 1702354 10.1002/adma.201702354. 29023971

[ref3] Chen X.; Korblova E.; Dong D.; Wei X.; Shao R.; Radzihovsky L.; Glaser M. A.; Maclennan J. E.; Bedrov D.; Walba D. M.; Clark N. A. First-Principles Experimental Demonstration of Ferroelectricity in a Thermotropic Nematic Liquid Crystal: Polar Domains and Striking Electro-Optics. Proc. Natl. Acad. Sci. U. S. A. 2020, 117 (25), 14021–14031. 10.1073/pnas.2002290117. 32522878 PMC7322023

[ref4] Manabe A.; Bremer M.; Kraska M. Ferroelectric Nematic Phase at and below Room Temperature. Liq. Cryst. 2021, 48 (8), 1079–1086. 10.1080/02678292.2021.1921867.

[ref5] Song Y.; Deng M.; Wang Z.; Li J.; Lei H.; Wan Z.; Xia R.; Aya S.; Huang M. Emerging Ferroelectric Uniaxial Lamellar (Smectic AF) Fluids for Bistable In-Plane Polarization Memory. J. Phys. Chem. Lett. 2022, 13 (42), 9983–9990. 10.1021/acs.jpclett.2c02846. 36263973

[ref6] Kikuchi H.; Matsukizono H.; Iwamatsu K.; Endo S.; Anan S.; Okumura Y. Fluid Layered Ferroelectrics with Global C_∞v_ Symmetry. Adv. Sci. 2022, 9 (26), 2202048 10.1002/advs.202202048. PMC9475520 35869031

[ref7] Chen X.; Martinez V.; Nacke P.; Korblova E.; Manabe A.; Klasen-Memmer M.; Freychet G.; Zhernenkov M.; Glaser M. A.; Radzihovsky L.; Maclennan J. E.; Walba D. M.; Bremer M.; Giesselmann F.; Clark N. A. Observation of a Uniaxial Ferroelectric Smectic A Phase. Proc. Natl. Acad. Sci. U. S. A. 2022, 119 (47), e2210062119 10.1073/pnas.2210062119. 36375062 PMC9704750

[ref8] Yang C.; Ye F.; Huang X.; Li J.; Zhang X.; Song Y.; Aya S.; Huang M. Fluorinated Liquid Crystals and Their Mixtures Giving Polar Phases with Enhanced Low-Temperature Stability. Liq. Cryst. 2024, 51 (4), 558–568. 10.1080/02678292.2024.2306309.

[ref9] Matsukizono H.; Sakamoto Y.; Okumura Y.; Kikuchi H. Exploring the Impact of Linkage Structure in Ferroelectric Nematic and Smectic Liquid Crystals. J. Phys. Chem. Lett. 2024, 15 (15), 4212–4217. 10.1021/acs.jpclett.3c03492. 38599584 PMC11033931

[ref10] Hobbs J.; Gibb C. J.; Mandle R. J. Emergent Antiferroelectric Ordering and the Coupling of Liquid Crystalline and Polar Order. Small Sci. 2024, 4, 2400189 10.1002/smsc.202400189. PMC11935032 40212248

[ref11] Nishikawa H.; Okada D.; Kwaria D.; Nihonyanagi A.; Kuwayama M.; Hoshino M.; Araoka F. Emergent Ferroelectric Nematic and Heliconical Ferroelectric Nematic States in an Achiral “Straight” Polar Rod Mesogen. Adv. Sci. 2024, 11, 2405718 10.1002/advs.202405718. PMC11633337 39099380

[ref12] Hobbs J.; Gibb C. J.; Pociecha D.; Szydłowska J.; Górecka E.; Mandle R. J. Polar Order in a Fluid Like Ferroelectric with a Tilted Lamellar Structure – Observation of a Polar Smectic C (SmC_P_) Phase. Angew. Chem. Int. Ed. 2025, 64, e202416545 10.1002/anie.202416545. PMC11753598 39475205

[ref13] Kikuchi H.; Nishikawa H.; Matsukizono H.; Iino S.; Sugiyama T.; Ishioka T.; Okumura Y. Ferroelectric Smectic C Liquid Crystal Phase with Spontaneous Polarization in the Direction of the Director. Adv. Sci. 2024, 11 (45), 2409827 10.1002/advs.202409827. PMC11615755 39439242

[ref14] Strachan G. J.; Górecka E.; Szydłowska J.; Makal A.; Pociecha D. Nematic and Smectic Phases with Proper Ferroelectric Order. Adv. Sci. 2025, 12, e2409754 10.1002/advs.202409754. PMC11744557 39585773

[ref15] Karcz J.; Herman J.; Rychłowicz N.; Kula P.; Górecka E.; Szydlowska J.; Majewski P. W.; Pociecha D. Spontaneous Chiral Symmetry Breaking in Polar Fluid–Heliconical Ferroelectric Nematic Phase. Science 2024, 384 (6700), 1096–1099. 10.1126/science.adn6812. 38843325

[ref16] Gibb C. J.; Hobbs J.; Nikolova D. I.; Raistrick T.; Berrow S. R.; Mertelj A.; Osterman N.; Sebastián N.; Gleeson H. F.; Mandle R. J. Spontaneous Symmetry Breaking in Polar Fluids. Nat. Commun. 2024, 15 (1), 5845 10.1038/s41467-024-50230-2. 38992039 PMC11239904

[ref17] Hird M. Fluorinated Liquid Crystals – Properties and Applications. Chem. Soc. Rev. 2007, 36 (12), 2070–2095. 10.1039/b610738a. 17982522

[ref18] Chen X.; Martinez V.; Korblova E.; Freychet G.; Zhernenkov M.; Glaser M. A.; Wang C.; Zhu C.; Radzihovsky L.; Maclennan J. E.; Walba D. M.; Clark N. A. The Smectic Z_A_ Phase: Antiferroelectric Smectic Order as a Prelude to the Ferroelectric Nematic. Proc. Natl. Acad. Sci. U. S. A. 2023, 120 (8), e2217150120 10.1073/pnas.2217150120. 36791101 PMC9974471

[ref19] Kumari P.; Basnet B.; Wang H.; Lavrentovich O. D. Ferroelectric Nematic Liquids with Conics. Nat. Commun. 2023, 14 (1), 748 10.1038/s41467-023-36326-1. 36765061 PMC9918734

[ref20] Clark N. A.; Chen X.; MacLennan J. E.; Glaser M. A. Dielectric Spectroscopy of Ferroelectric Nematic Liquid Crystals: Measuring the Capacitance of Insulating Interfacial Layers. Phys. Rev. Res. 2024, 6 (1), 013195 10.1103/PhysRevResearch.6.013195.

[ref21] Vaupotič N.; Krajnc T.; Gorecka E.; Pociecha D.; Matko V. Ferroelectric Nematics: Materials with High Permittivity or Low Resistivity? arXiv November 2, 2024.

[ref22] Matko V.; Gorecka E.; Pociecha D.; Matraszek J.; Vaupotič N. Interpretation of Dielectric Spectroscopy Measurements of Ferroelectric Nematic Liquid Crystals. Phys. Rev. Res. 2024, 6 (4), L042017 10.1103/PhysRevResearch.6.L042017.

[ref23] Barthakur A.; Karcz J.; Kula P.; Dhara S. Critical Splay Fluctuations and Colossal Flexoelectric Effect above the Nonpolar to Polar Nematic Phase Transition. Phys. Rev. Mater. 2023, 7 (3), 035603 10.1103/PhysRevMaterials.7.035603.

[ref24] Gramsbergen E. F.; de Jeu W. H. First- and Second-Order Smectic-A to Nematic Phase Transitions in p,P′-Dialkylazoxybenzenes Studied by Birefringence. J. Chem. Soc. Faraday Trans. 2 Mol. Chem. Phys. 1988, 84 (8), 1015–1021. 10.1039/F29888401015.

[ref25] Li J.; Nishikawa H.; Kougo J.; Zhou J.; Dai S.; Tang W.; Zhao X.; Hisai Y.; Huang M.; Aya S. Development of Ferroelectric Nematic Fluids with Giant-ε Dielectricity and Nonlinear Optical Properties. Sci. Adv. 2021, 7 (17), eabf5047 10.1126/sciadv.abf5047. 33883139 PMC8059932

[ref26] Cruickshank E. The Emergence of a Polar Nematic Phase: A Chemist’s Insight into the Ferroelectric Nematic Phase. ChemPlusChem. 2024, 89 (5), e202300726 10.1002/cplu.202300726. 38452282

[ref27] Gibb C. J.; Hobbs J.; Mandle R. J. Systematic Fluorination Is a Powerful Design Strategy Towards Fluid Molecular Ferroelectrics. J. Am. Chem. Soc. 2025, 147, 4571–4577. 39853340 10.1021/jacs.4c16555 PMC11803714

