# Supplementary material for: Fluorination: Simple Change but Complex Impact on Ferroelectric Nematic and Smectic Liquid Crystal Phases
Source: J Am Chem Soc. 2025 Feb 7;147(7):6058–66. doi: 10.1021/jacs.4c16802 (PMC11848818; doi:10.1021/jacs.4c16802)
Supplement: Supplementary file 1 — ja4c16802_si_001.pdf [file ja4c16802_si_001.pdf]

# Fluorination: Simple Change but Complex Impact on Ferroelectric Nematic and Smectic Liquid Crystal Phases

Grant J. Strachan,[1] Ewa Górecka,[1] Jordan Hobbs,[2] and Damian Pociecha.[1]

[1] Faculty of Chemistry, University of Warsaw, ul. Pasteura 1, 02-093 Warsaw, Poland

[2] School of Physics and Astronomy, University of Leeds, Leeds, UK, LS2 9JT

*Author for correspondence: g.strachan@chem.uw.edu.pl

**Experimental Methods** ........ 1

**Synthetic Procedures and Structural Characterisation** ........ 2

**NMR Spectra** ........ 12

**Additional results related to structure of observed mesophases** ........ 20

**References** ........ 23

## Experimental Methods

Transition temperatures and the associated enthalpy changes were measured by differential scanning calorimetry using a TA DSC Q200 instrument. Measurements were performed under a nitrogen atmosphere with a heating/cooling rate of 10 K $min^{-1}$, unless otherwise specified.

Observations of optical textures of liquid crystalline phases was carried out by polarised-light optical microscopy using a Zeiss AxioImager.A2m microscope equipped with a Linkam heating stage.

Optical birefringence was measured with a setup based on a photoelastic modulator (PEM-90, Hinds) working at a modulation frequency $f$ = 50 kHz; as a light source a halogen lamp (Hamamatsu LC8) equipped with narrow bandpass filters was used. The transmitted light intensity was monitored with a photodiode (FLC Electronics PIN-20) and the signal was deconvoluted with a lock-in amplifier (EG&G 7265) into 1$f$ and 2$f$ components to yield a retardation induced by the sample. Knowing the sample thickness, the retardation was recalculated into optical birefringence. Samples were prepared in 1.6-μm-thick cells with planar anchoring. The alignment quality was checked prior to measurement by inspection under the polarised-light optical microscope.

X-ray diffraction measurements of samples in liquid crystalline phases were carried out using a Bruker D8 GADDS system, equipped with micro-focus-type X-ray source with Cu anode and dedicated optics and VANTEC2000 area detector. Small angle diffraction experiments were performed on a Bruker Nanostar system (IμS microfocus source with copper target, MRI heating stage, Vantec 2000 area detector).

Spontaneous electric polarisation was determined by integration of the current peaks recorded during polarization switching upon applying a triangular-wave voltage. 5-μm-thick cells with ITO or gold electrodes and no polymer aligning layers were used, and the switching current was determined by recording the voltage drop on a resistor connected in series with the sample.

The complex dielectric permittivity, ε*, was measured using a Solartron 1260 impedance analyser, in the 1 Hz −10 MHz frequency range, and a probe voltage of 50 mV. The material

was placed in a 5- or 10-μm-thick glass cell with gold electrodes. Cells without polymer aligning layers were used, as the presence of the thin (~10 nm) polyimide layers at the cell surfaces acts as an additional high capacitance capacitor in a series circuit with the capacitor filled with the LC sample, which for materials with very high values of permittivity, may strongly affect the measured permittivity of the LC phases. Lack of a surfactant layer resulted in a random configuration of the director in the LC phases.

Light diffraction studies were performed for the samples placed on a heating stage and illuminated from below with a green (520 nm) laser. The diffraction pattern was recorded on the screen placed above the sample, the angular position of the observed diffraction signal allowed for calculation of the related periodicity of the stripe pattern in the sample and thus the helical pitch length.

DFT geometry optimization was carried out at the B3LYP-GD3BJ/cc-pVTZ level of theory using Gaussian 16 (Revision C.01)[1] on the Ares cluster of the Polish high-performance computing infrastructure PLGrid (HPC Center: ACK Cyfronet AGH. Following geometry optimization, a frequency calculation was used to confirm that the obtained structure was at an energy minimum. Generation of 1D representations of the electrostatic potential was carried out according to previously reported methods[2,3]. In brief, the 3D ESP surface was reduced by reading the gaussian generated cube file for 3D electron density and finding a contour through 2D slices along the molecule where the electron density isovalue equal 0.0004. We then find the values of electrostatic potential that fall along these contours and then average them where the average forms the value of ESP at that point along the z-axis. Each average ESP value along the Z-axis is scaled by the length of the contour.

## Synthetic Procedures and Structural Characterisation

Unless otherwise stated, all materials were obtained from commercial sources and used without further purification. Reactions were monitored using thin layer chromatography (TLC) using aluminium-backed plates with a coating of Merck Kieselgel 60 F254 silica and an appropriate solvent system. Spots were visualised using UV light (254 nm). Flash column chromatography was carried out using silica grade 60 Å 40-63 micron. $^{1}$H, $^{19}$F, and $^{13}$C NMR spectra were recorded on a 400 MHz Agilent NMR spectrometer using either $CDCl_3$ or DMSO-$d_6$ as solvent and using residual non-deuterated trace solvents as reference. Chemical shifts (δ) are given in ppm relative to TMS (δ = 0.00 ppm). Mass spectroscopy was conducted on a Micromass LCT instrument.

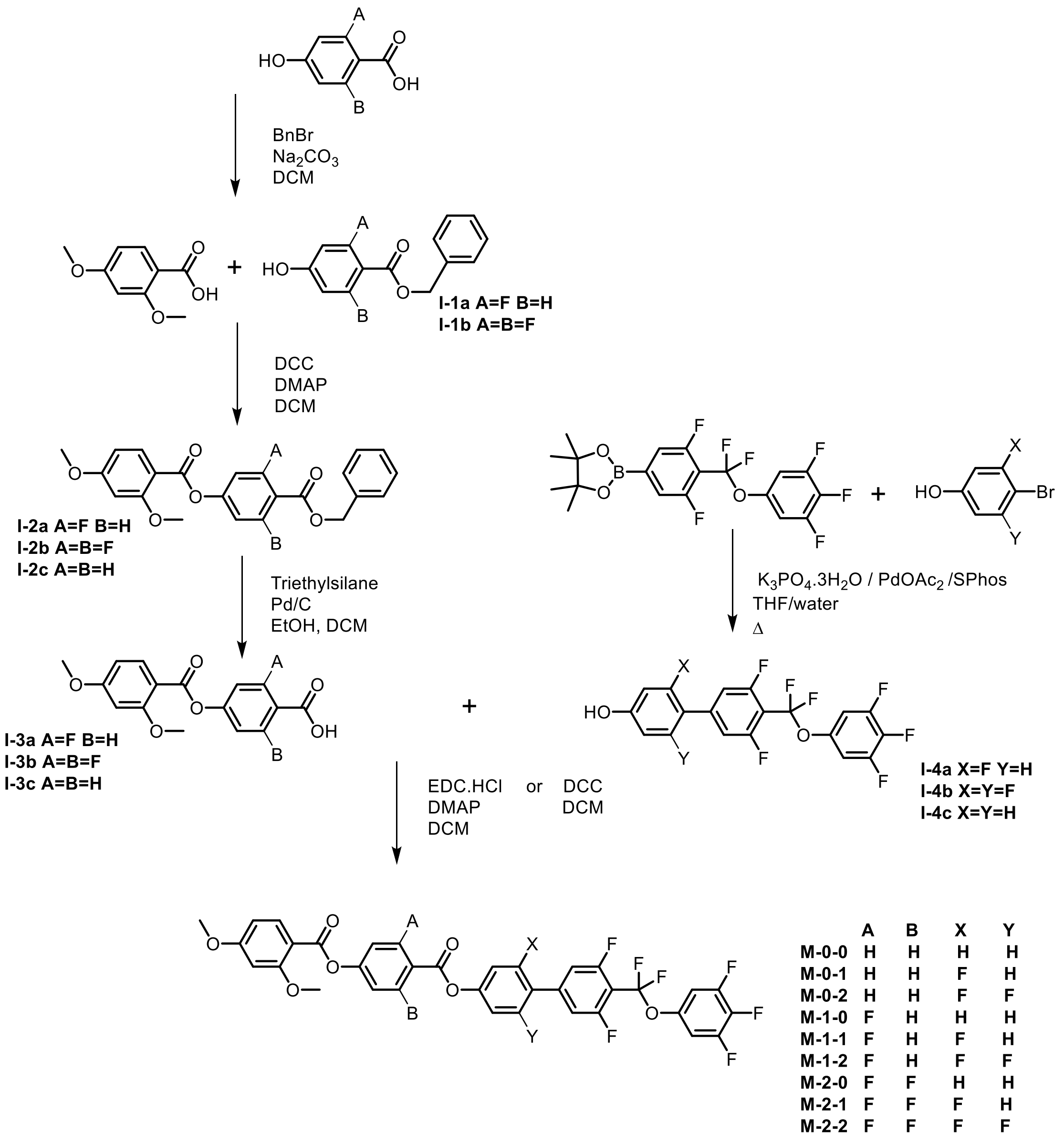


| | A | B | X | Y |
|---|---|---|---|---|
| M-0-0 | H | H | H | H |
| M-0-1 | H | H | F | H |
| M-0-2 | H | H | F | F |
| M-1-0 | F | H | H | H |
| M-1-1 | F | H | F | H |
| M-1-2 | F | H | F | F |
| M-2-0 | F | F | H | H |
| M-2-1 | F | F | F | H |
| M-2-2 | F | F | F | F |

*Scheme 1: Synthetic route to the new materials reported here.*

The synthesis of 2-(4-(difluoro(3,4,5-trifluorophenoxy)methyl)-3,5-difluorophenyl)-4,4,5,5-tetramethyl 1,3,2-dioxaborolane, intermediate compounds **I-2c**, **I-3c,** and **I-4b**, and the liquid crystal **M-0-1** has been reported previously.[4]

## Benzyl ester protection

The corresponding fluorinated 4-hydroxybenzoic acid and $Na_2CO_3$ were stirred in dry DMF under an argon atmosphere. Benzyl bromide was added and the reaction stirred at room temperature overnight. The reaction mixture was added to water and acidified to approximately pH 4 using 1 M HCl, then extracted 3x with diethyl ether. The organics were combined, dried over magnesium sulfate and the solvent removed under vacuum. The crude product thus obtained was recrystalised from toluene.

**I-1a**:

2-fluoro-4-hydroxybenzoic acid 1.514 g, 10 mmol, 1 eq.

| | |
|---|---|
| $Na_2CO_3$ | 1.092 g, 10 mmol, 1 eq. |
| Benzylbromide | 1.2 ml, 1.73 g, 10 mmol, 1 eq. |
| DMF | 12 ml |

Yield 1.14 g

$^1$H NMR (400 MHz, $CDCl_3$) δ 7.89 (t, J = 8.4 Hz, 1H), 7.48 – 7.27 (m, 5H), 6.67 – 6.57 (m, 2H), 5.78 (s, 1H), 5.35 (s, 2H).

$^{13}$C NMR (101 MHz, $CDCl_3$) δ 165.00, 164.38, 164.34, 162.40, 161.65, 161.52, 135.80, 133.78, 133.75, 128.57, 128.21, 128.00, 111.55, 111.52, 110.65, 110.56, 104.31, 104.06, 66.75.

**I-1b**:

| | |
|---|---|
| 2,6-difluoro-4-hydroxybenzoic acid | 1.74 g, 10 mmol, 1 eq. |
| $Na_2CO_3$ | 1.092 g, 10 mmol, 1 eq. |
| Benzylbromide | 1.2 ml, 1.73 g, 10 mmol, 1 eq. |
| DMF | 12 ml |

Yield 1.22 g

$^1$H NMR (400 MHz, $CDCl_3$) δ 7.46 – 7.30 (m, 5H), 6.46 – 6.39 (m, 2H), 5.73 (s, 1H), 5.36 (s, 2H).

$^{13}$C NMR (101 MHz, $CDCl_3$) δ 163.87, 162.13, 161.81, 161.31, 161.12, 160.36, 160.25, 160.18, 135.36, 128.57, 128.28, 128.06, 100.33, 100.04, 67.27.

**Esterification I-2**

2,4-dimethoxy benzoic acid and EDC.HCl were dissolved in DCM and stirred for 10 minutes. The appropriate benzyl 4-hydroxybenzoate and 4-dimethylaminopyridine (DMAP) were added and the reaction was left stirring at room temperature overnight. The reaction was washed 3x with water and the solvent removed *in vacuo*. The crude product was recrystallised from ethanol to yield the product as a white solid.

**I-2a**

| | |
|---|---|
| 2-fluoro-4-hydroxybenzyl benzoate | 400 mg, 1.6 mmol, 1 eq. |
| 2,4-dimethoxybenzoic acid | 326 mg, 1.8 mmol, 1.1 eq. |
| EDC.HCl | 629 mg, 3.3 mmol, 2 eq. |
| DMAP | 19 mg, 0.1 mmol, 0.1 eq. |
| DCM | 15 ml |

Yield 346 mg

$^{1}$H NMR (400 MHz, $CDCl_3$) δ 8.08 – 7.97 (m, *J* = 15.3, 8.3 Hz, 2H), 7.49 – 7.30 (m, 5H), 7.09 (d, *J* = 9.9 Hz, 2H), 6.59 – 6.50 (m, 2H), 5.39 (s, 2H), 3.92 (s, 3H), 3.89 (s, 3H).

$^{13}$C NMR (101 MHz, $CDCl_3$) δ 171.56, 165.41, 163.78, 163.68, 163.64, 162.53, 162.37, 161.18, 155.70, 155.59, 135.72, 134.63, 132.87, 128.58, 128.23, 128.07, 117.83, 117.80, 115.69, 115.60, 111.37, 111.11, 110.08, 109.99, 104.94, 98.96, 66.91, 56.02, 55.62.

**I-2b**

| | |
|---|---|
| 2,6-difluoro-4-hydroxybenzyl benzoate | 1.056 g, 4 mmol, 1 eq. |
| 2,4-dimethoxybenzoic acid | 817 mg, 4.4 mmol, 1.1 eq. |
| EDC.HCl | 1.188 g, 6 mmol, 1.5 eq. |
| DMAP | 49 mg, 0.4 mmol, 0.1 eq. |
| DCM | 25 ml |

Yield 1.5 g

$^{1}$H NMR (400 MHz, $CDCl_3$) δ 8.02 (d, *J* = 8.8 Hz, 1H), 7.50 – 7.28 (m, *J* = 19.5, 16.9, 7.1 Hz, 5H), 6.91 (d, *J* = 9.4 Hz, 2H), 6.56 (d, *J* = 8.8 Hz, 1H), 6.52 (s, 1H), 5.39 (s, 2H), 3.92 (s, 3H), 3.89 (s, 3H).

$^{13}$C NMR (101 MHz, $CDCl_3$) δ 165.58, 162.62, 162.53, 162.49, 161.95, 161.04, 160.01, 159.93, 154.46, 154.30, 154.18, 135.15, 134.66, 128.59, 128.35, 128.14, 109.65, 106.94, 106.69, 105.02, 98.95, 67.52, 56.01, 55.64.

**Deprotection I-3**

Under an argon atmosphere triethylsilane was added dropwise to a stirred solution of **I-2x** and 5 % Pd/C in a 1:1 mix of ethanol and DCM. The reaction was stirred for 5 minutes after addition was complete, then filtered through celite and the solvent removed *in vacuo*. The crude product was washed with hexane to yield the product as a white powder.

**I-3a**

| | |
|---|---|
| **I-2a** | 405 mg, 1 mmol |
| 5% Pd/C | 130 mg |
| Triethylsilane | 3 ml |

DCM 5 ml

EtOH 5 ml

Yield 290 mg

$^{1}$H NMR (400 MHz, $CDCl_3$) δ 8.11 – 8.02 (m, *J* = 14.5, 8.8 Hz, 1H), 7.16 – 7.10 (m, 1H), 6.57 (d, *J* = 8.9 Hz, 1H), 6.54 (s, 1H), 3.93 (s, 2H), 3.90 (s, 2H).

$^{13}C$ NMR (101 MHz, DMSO-$d_6$) δ 165.53, 164.94, 164.91, 163.18, 162.44, 162.33, 160.61, 155.35, 155.23, 134.62, 133.21, 133.19, 118.92, 118.89, 117.24, 117.14, 111.95, 111.70, 109.81, 106.18, 99.41, 56.45, 56.19.

**I-3b**

| | |
|---|---|
| **I-2b** | 1.175 g, 2.8 mmol |
| 5% Pd/C | 240 mg |
| Triethylsilane | 4.5 ml |

DCM 7 ml

EtOH 8 ml

Yield 720 mg

$^{1}H$ NMR (400 MHz, $CDCl_3$) δ 8.03 (d, *J* = 8.8 Hz, 1H), 6.95 (d, *J* = 10.1 Hz, 2H), 6.57 (d, *J* = 8.9 Hz, 1H), 6.53 (s, 1H), 3.93 (s, 3H), 3.90 (s, 3H).

$^{13}C$ NMR (101 MHz, DMSO-$d_6$) δ 165.66, 162.48, 162.22, 162.09, 161.35, 161.25, 158.84, 158.75, 153.74, 134.74, 109.43, 108.04, 108.01, 107.78, 107.75, 106.22, 99.38, 56.45, 56.21.

**Suzuki coupling I-4**

2-(4-(difluoro(3,4,5-trifluorophenoxy)methyl)-3,5-difluorophenyl)-4,4,5,5-tetramethyl 1,3,2-dioxaborolane, the appropriate 4-bromophenol, and base were dissolved in THF (20 ml) and distilled water (1 ml), sparged with argon, and refluxed for 1 hour. Palladium acetate and S-Phos were added, and the reaction was refluxed for 4 hours. The mixture was cooled to RT, acidified with 1 M HCl, extracted with three portions of DCM and the organic layers were combined, dried over magnesium sulfate and the solvent removed *in vacuo*. The crude product was purified via column chromatography.

**I-4a**

| | |
|---|---|
| Boronic ester | 957 mg, 2.2 mmol, 1.1 eq. |
| 4-bromophenol | 346 mg, 2 mmol, 1 eq. |
| $K_3PO_4.3H_2O$ | 1.862 g, 7 mmol, 3.5 eq |
| $Pd(OAc)_2$ | 29 mg, 5 mol% |
| Sphos | 88 mg, 10 mol% |
| Yield | 320 mg |
| $R_f$ (DCM) | 0.4 |

$^{1}H$ NMR (400 MHz, $CDCl_3$) δ 7.47 (d, *J* = 8.5 Hz, 2H), 7.16 (d, *J* = 10.9 Hz, 2H), 7.05 – 6.88 (m, 4H), 5.12 (s, 1H).

$^{19}$F NMR (376 MHz, $CDCl_3$) δ -61.57 (t, 2F, *J* = 26.1 Hz), -110.72 (td, 2F, *J* = 26.1, 12.2 Hz), -132.36 – -133.83 (m, 2F,), -163.26 (ddd, 1F, *J* = 20.7, 15.8, 5.3 Hz).

$^{13}$C NMR (101 MHz, $CDCl_3$) δ 161.51, 161.45, 159.00, 158.92, 156.71, 152.28, 152.22, 152.17, 152.12, 149.78, 149.73, 149.64, 149.62, 146.57, 146.46, 146.41, 146.36, 144.73, 144.70, 144.59, 139.77, 139.62, 139.45, 137.29, 137.19, 137.13, 136.98, 130.02, 128.40, 122.99, 120.31, 117.66, 116.09, 110.42, 110.39, 110.18, 110.15, 107.52, 107.46, 107.28.

**I-4c**

| | |
|---|---|
| Boronic ester | 1.12 g, 2.5 mmol, 1.1 eq. |
| 2,6-difluoro-4-bromophenol | 475 mg mg, 2.25 mmol, 1 eq. |
| $K_2CO_3$ | 1.66 g, (2M aq. solution) |
| $Pd(OAc)_2$ | 39 mg, 5 mol% |
| Sphos | 100 mg, 10 mol% |
| Yield | 780 mg |
| $R_f$ (DCM) | 0.26 |

$^{1}$H NMR (400 MHz, $CDCl_3$) δ 7.11 (d, *J* = 10.8 Hz, 2H), 7.05 – 6.93 (m, 2H), 6.53 (d, *J* = 9.2 Hz, 2H), 5.63 (s, 1H).

$^{19}$F NMR (376 MHz, $CDCl_3$) δ -61.92 (t, 2F, *J* = 26.4 Hz, 2), -111.43 (td, 2F, *J* = 26.4, 11.1 Hz), -113.18 (d, 2F, *J* = 9.3 Hz), -132.68 (dd, 2F, *J* = 20.6, 8.1 Hz), -163.36 (t, 1F, *J* = 20.6 Hz).

$^{13}$C NMR (101 MHz, $CDCl_3$) δ 161.59, 161.50, 160.90, 160.84, 159.11, 159.01, 158.35, 158.29, 157.80, 157.66, 157.51, 152.29, 152.24, 152.19, 152.15, 152.13, 149.79, 149.74, 149.68, 149.63, 144.68, 144.64, 144.54, 144.53, 144.45, 144.40, 139.83, 139.68, 139.51, 137.34, 137.19, 137.04, 135.30, 135.19, 135.08, 122.75, 122.73, 120.09, 117.45, 117.44, 114.67, 114.46, 114.43, 107.59, 107.53, 107.43, 107.36, 100.24, 100.16, 100.04, 99.97, 99.95.

**Esterification M-X-X**

Method A

Intermediate acid **I-3x** and EDC.HCl were dissolved in 10 ml DCM and stirred for 5 minutes. The appropriate phenol and DMAP were added, and the mixture stirred overnight and monitored by TLC. The reaction mixture was washed 3 times with water and the organic layer dried over magnesium sulfate and removed *in vacuo*. The crude solid was purified by column chromatography (gradient elution, 50/50 DCM: hexane → DCM) and the solid thus obtained was dissolved in the minimum amount of chloroform and precipitated with hexane.

Method B

Intermediate acid **I-3x** and DCC were dissolved in 10 ml DCM in an icebath and stirred for 30 minutes. The appropriate phenol was added, and the mixture stirred overnight and monitored by TLC. The reaction mixture was filtered and the solvent removed *in vacuo*. The crude solid was purified by column chromatography (gradient elution, 50/50 DCM: hexane → DCM) and the solid thus obtained was dissolved in the minimum amount of chloroform and precipitated with hexane

| | Method | EDC.HCl | DCC | DMAP | Acid | Phenol | Yield |
|---|---|---|---|---|---|---|---|
| **M-0-0** | B | - | 45 mg, 0.2 mmol | - | 86 mg, 0.3 mmol | 86 mg, 0.2 mmol | 46 mg |
| **M-0-1** | Previously reported[4] | | | | | | |
| **M-0-2** | A | 46 mg, 0.24 mmol | - | 6 mg, 0.05 mmol | 62 mg, 0.2 mmol | 70 mg, 0.17 mmol | 37 mg |
| **M-1-0** | B | - | 71 mg, 0.3 mmol | - | 80 mg, 0.25 mmol | 76 mg, 1.9 mmol | 45 mg |
| **M-1-1** | A | 51 mg, 0.27 mmol | - | 6 mg, 0.05 mmol | 83 mg, 0.18 mmol | 67 mg, 0.16 mmol | 53 mg |
| **M-1-2** | A | 310 mg, 1.6 mmol | - | 20 mg, 0.16 mmol | 199 mg, 0.63 mmol | 242 mg, 9.57 mmol | 92 mg |
| **M-2-0** | B | - | 32 mg, 0.16 mmol | - | 70 mg, 0.2 mmol | 64 mg, 0.16 mmol | 23 mg |
| **M-2-1** | B | - | 32 mg, 0.16 mmol | - | 70 mg, 0.21 mmol | 65 mg, 0.15 mmol | 30 mg |
| **M-2-2** | B | - | 41 mg, 0.2 mmol | - | 89 mg, 0.26 mmol | 90 mg, 0.2 mmol | 75 mg |

**M-0-0**

HRMS (ESI) m/z Calculated for $C_{35}H_{21}O_7F_7$:
$[M+Na]^+$ theoretical mass: 709.10677, found 709.10584, difference -1.3 ppm.

$^1$H NMR (400 MHz, $CDCl_3$) δ 8.28 (d, *J* = 8.5 Hz, 2H), 8.11 (d, *J* = 8.4 Hz, 1H), 7.64 (d, *J* = 8.4 Hz, 2H), 7.37 (overlapping doublets, 4H), 7.23 (d, $J_{HF}$ = 10.8 Hz, 2H), 7.03 – 6.97 (m, 2H), 6.58 (d, *J* = 8.4 Hz, 1H), 6.55 (s, 1H), 3.95 (s, 3H), 3.91 (s, 3H).

$^{19}$F NMR (376 MHz, $CDCl_3$) δ -61.70 (t, 2F, *J* = 26.2 Hz), -110.16 (td, 2F, *J* = 26.2, $J_{HF}$ = 10.8 Hz), -132.48 (dd, 2F, *J* = 20.7, $J_{HF}$ = 7.8 Hz), -163.16 (t, 2F, *J* = 20.7 Hz).

$^{13}$C NMR (101 MHz, $CDCl_3$) δ 165.32, 164.37, 162.85, 162.48, 161.56, 161.48, 159.00, 158.94, 155.65, 152.30, 152.25, 152.19, 152.14, 151.85, 149.80, 149.75, 149.70, 149.65, 146.18, 146.08, 145.97, 144.65, 144.63, 139.82, 139.67, 139.49, 137.33, 137.17, 137.02, 135.13, 134.62, 131.75, 128.18, 126.20, 122.63, 122.36, 120.23, 117.55, 111.14, 111.11, 110.90, 110.87, 110.43, 108.29, 107.55, 107.49, 107.38, 107.32, 104.93, 98.99, 56.04, 55.62.

**M-0-2**

HRMS (ESI) m/z Calculated for $C_{35}H_{19}O_7F_9$:
$[M+H]^+$ theoretical mass: 723.10598, found 723.10541, difference -0.79 ppm.

$[M+Na]^+$ theoretical mass: 745.08793, found 745.08720, difference -0.98 ppm.

$^1$H NMR (400 MHz, $CDCl_3$) δ 8.25 (d, *J* = 6.9 Hz, 2H), 8.11 (d, *J* = 8.8 Hz, 1H), 7.39 (d, *J* = 6.9 Hz, 2H), 7.17 (d, $J_{HF}$ = 10.4 Hz, 2H), 7.02 (overlapping peaks *J* = 9.2 Hz, 4H), 6.58 (d, *J* = 8.8 Hz, 1H), 6.55 (s, 1H), 3.95 (s, 3H), 3.91 (s, 3H).

$^{19}$F NMR (376 MHz, $CDCl_3$) δ -61.98 (t, 2F, *J* = 26.5 Hz), -110.64 (td, 2F, *J* = 26.5, $J_{HF}$ = 10.4 Hz), -112.07 (d, 2F, $J_{HF}$ = 8.5 Hz), -132.46 (dd, 2F, *J* = 20.8, $J_{HF}$ = 8.0 Hz), -163.09 (tt, 1F, *J* = 20.8, 5.6 Hz).

$^{13}$C NMR (101 MHz, $CDCl_3$) δ 165.38, 163.57, 162.76, 162.52, 161.02, 160.97, 160.94, 160.89, 158.47, 158.39, 156.00, 152.31, 152.26, 152.19, 152.15, 152.05, 151.90, 149.82, 149.76, 149.71, 149.66, 139.87, 139.71, 139.57, 137.23, 134.63, 134.38, 134.28, 131.87, 125.37, 122.52, 119.99, 114.85, 114.60, 110.30, 107.62, 107.56, 107.45, 107.38, 106.95, 106.93, 106.87, 106.74, 106.68, 106.65, 104.96, 98.99, 56.04, 55.63.

**M-1-0**

HRMS (ESI) m/z Calculated for $C_{35}H_{20}O_7F_8$:
$[M+H]^+$ theoretical mass: 705.11540, found 705.11519, difference -0.31 ppm.
$[M+Na]^+$ theoretical mass: 727.09735, found 727.09669, difference -0.91 ppm.

$^1$H NMR (400 MHz, $CDCl_3$) δ 8.18 (t, $J_{HF}$ = 8.3 Hz, 1H), 8.08 (d, *J* = 8.6 Hz, 1H), 7.63 (d, *J* = 8.2 Hz, 2H), 7.38 (d, *J* = 8.1 Hz, 2H), 7.25 – 7.13 (m, 4H), 7.04 – 6.96 (m, 2H), 6.58 (d, *J* = 8.6 Hz, 1H), 6.55 (s, 1H), 3.95 (s, 3H), 3.91 (s, 3H).

$^{19}$F NMR (376 MHz, $CDCl_3$) δ -61.70 (t, 2F, *J* = 26.2 Hz), -104.67 (dd, 1F, *J* = 10.6, $J_{HF}$ = 8.3 Hz), -110.15 (td, 2F, *J* = 26.2, $J_{HF}$ = 10.6 Hz), -132.48 (dd, 2F, *J* = 20.8, $J_{HF}$ = 8.0 Hz), -163.17 (tt, 1F, *J* = 20.8, 5.8 Hz).

$^{13}$C NMR (101 MHz, $CDCl_3$) δ 165.54, 164.14, 162.63, 162.26, 162.06, 162.02, 161.52, 158.99, 158.94, 156.52, 156.41, 152.30, 152.25, 152.19, 152.13, 151.50, 149.80, 149.75, 149.70, 149.64, 146.14, 146.04, 145.93, 144.76, 144.73, 144.64, 144.61, 144.58, 137.32, 137.17, 137.02, 135.26, 134.67, 133.29, 133.28, 128.17, 122.59, 118.17, 118.13, 114.69, 114.59, 111.65, 111.39, 111.15, 111.12, 110.91, 110.88, 109.91, 108.63, 108.46, 108.31, 107.55, 107.49, 107.38, 107.31, 105.02, 98.98, 56.04, 55.64.

**M-1-1**

HRMS (ESI) m/z Calculated for $C_{35}H_{19}O_7F_9$:
$[M+H]^+$ theoretical mass: 723.10598, found 723.10515, difference -1.2 ppm.

$^1$H NMR (400 MHz, $CDCl_3$) δ 8.16 (t, $J_{HF}$ = 8.5, 1H), 8.08 (d, *J* = 8.9 Hz, 1H), 7.50 (t, $J_{HF}$ = 8.5, 1H), 7.20 (m, 6H), 7.00 (m, 2H), 6.58 (d, *J* = 8.9 Hz, 1H), 6.55 (s, 1H), 3.95 (s, *J* = 9.3 Hz, 3H), 3.91 (s, 3H).

$^{19}$F NMR (376 MHz, $CDCl_3$) δ -61.83 (t, 2F, *J* = 26.3 Hz), -104.40 (m, 1F), -110.43 (td, 2F *J* = 26.3, $J_{HF}$ = 11.2 Hz), -113.83 (m, 1F), -131.29 – -134.95 (m, 2F), -162.50 – -164.21 (m, 1F).

$^{13}$C NMR (101 MHz, $CDCl_3$) δ 165.57, 164.19, 162.65, 162.22, 161.64, 161.60, 161.57, 161.22, 161.16, 160.75, 158.65, 158.59, 158.24, 156.73, 156.61, 152.30, 152.19, 152.14, 152.01, 151.89, 149.80, 149.76, 149.70, 144.55, 140.82, 140.72, 140.62, 139.68, 137.20,

134.68, 133.30, 130.55, 123.38, 123.24, 122.79, 122.71, 120.10, 118.46, 118.42, 118.24, 118.21, 114.28, 114.18, 113.21, 112.97, 111.70, 111.45, 111.04, 110.78, 109.84, 107.58, 107.51, 107.40, 107.34, 105.04, 98.98, 56.04, 55.64.

**M-1-2**

HRMS (ESI) m/z Calculated for $C_{35}H_{18}O_7F_{10}$:
$[M+H]^+$ theoretical mass: 741.09656, found 741.09605, difference -0.69 ppm.

$^1$H NMR (400 MHz, $CDCl_3$) δ 8.15 (t, $J_{HF}$ = 8.5 Hz, 1H), 8.08 (d, $J$ = 8.8 Hz, 1H), 7.22 – 7.14 (m, 4H), 7.02 (overlapping doublet and triplet, 4H), 6.58 (d, $J$ = 8.8 Hz, 1H), 6.55 (s, 1H), 3.95 (s, 3H), 3.91 (s, 3H).

$^{19}$F NMR (376 MHz, $CDCl_3$) δ -62.01 (t, 2F, $J$ = 26.5 Hz), -103.23 – -105.40 (1F, m), -110.63 (td, 2F, $J$ = 26.5, $J_{HF}$ = 10.4 Hz), -112.00 (d, 2F $J_{HF}$ = 8.5 Hz), -132.45 (dd, 2F, $J$ = 20.8, $J_{HF}$ = 8.0 Hz), -163.07 (tt, 1F, $J$ = 20.8, 5.7 Hz).

$^{13}$C NMR (101 MHz, $CDCl_3$) δ 165.60, 164.23, 162.67, 162.17, 161.60, 161.26, 161.22, 161.01, 160.95, 160.87, 158.45, 158.36, 156.93, 156.81, 152.30, 152.25, 152.20, 152.14, 151.78, 151.63, 151.49, 150.85, 150.79, 149.81, 149.75, 149.70, 149.65, 144.49, 139.85, 139.70, 137.38, 137.23, 137.05, 134.70, 134.45, 134.33, 134.21, 133.34, 122.65, 119.98, 118.34, 118.30, 114.85, 114.60, 113.88, 113.79, 113.20, 113.01, 111.77, 111.52, 109.74, 107.64, 107.57, 107.46, 107.40, 106.94, 106.92, 106.86, 106.73, 106.67, 106.64, 105.04, 98.96, 56.04, 55.66.

**M-2-0**

HRMS (ESI) m/z Calculated for $C_{35}H_{19}O_7F_9$:
$[M+Na]^+$ theoretical mass: 745.08793, found 745.08738, difference -0.73 ppm.

$^1$H NMR (400 MHz, $CDCl_3$) δ 8.05 (d, $J$ = 8.7 Hz, 1H), 7.63 (d, $J$ = 8.5 Hz, 2H), 7.40 (d, $J$ = 8.5 Hz, 2H), 7.26 – 7.18 (m, 2H), 7.05 – 6.95 (m, 4H), 6.58 (dd, $J$ = 8.7, 2.1 Hz, 1H), 6.54 (d, $J$ = 2.1 Hz, 1H), 3.95 (s, 3H), 3.91 (s, 3H).

$^{19}$F NMR (376 MHz, $CDCl_3$) δ -61.71 (t, 2F, $J$ = 26.2 Hz), -107.16 (d, 2F, $J_{HF}$ = 9.5 Hz), -110.09 (td, 2F, $J$ = 26.4, $J_{HF}$ = 10.8 Hz), -132.48 (dd, 2F, $J$ = 20.8, $J_{HF}$ = 8.1 Hz), -163.16 (tt, 1F, $J$ = 20.7, 5.7 Hz).

$^{13}$C NMR (101 MHz, $CDCl_3$) δ 165.70, 162.90, 162.80, 162.72, 161.86, 161.58, 161.51, 160.32, 160.25, 159.34, 159.02, 158.99, 158.94, 155.28, 155.14, 154.99, 152.30, 152.25, 152.20, 152.15, 152.14, 151.21, 149.81, 149.76, 149.69, 149.63, 146.07, 145.96, 145.86, 144.61, 135.53, 134.71, 128.23, 122.49, 111.18, 111.16, 110.94, 110.92, 109.50, 107.55, 107.49, 107.37, 107.31, 107.21, 107.17, 106.96, 106.92, 105.10, 98.97, 56.04, 55.66.

**M-2-1**

HRMS (ESI) m/z Calculated for $C_{35}H_{18}O_7F_{10}$:
$[M+H]^+$ theoretical mass: 741.09656, found 741.09588, difference -0.92 ppm.

$^1H$ NMR (400 MHz, $CDCl_3$) δ 8.05 (d, $J$ = 8.7 Hz, 1H), 7.50 (t, $J$ = 8.0 Hz, 1H), 7.24 – 7.17 (m, 4H), 7.05 – 6.96 (m, 4H), 6.58 (d, $J$ = 8.7 Hz, 1H), 6.54 (s, 1H), 3.95 (s, 3H), 3.91 (s, 3H).

$^{19}F$ NMR (376 MHz, $CDCl_3$) δ -61.84 (t, 2F, $J$ = 26.4 Hz), -106.87 (d, 2F, $J_{HF}$ = 9.5 Hz), -110.38 (td, 2F, $J$ = 26.4, $J_{HF}$ = 10.6 Hz), -113.64 (t, 1F, $J_{HF}$ = 9.6 Hz), -132.46 (dd, 2F, $J$ = 20.8, $J_{HF}$ = 8.0 Hz), -163.12 (tt, 1F, $J$ = 20.8, 5.7 Hz).

$^{13}C$ NMR (101 MHz, $CDCl_3$) δ 165.74, 162.98, 162.90, 162.74, 161.82, 161.22, 161.18, 160.74, 160.40, 160.32, 158.93, 158.91, 158.89, 158.64, 158.61, 158.23, 155.52, 155.39, 155.24, 152.31, 152.25, 152.20, 152.15, 151.65, 151.54, 149.81, 149.76, 149.69, 149.65, 140.75, 140.62, 140.48, 139.67, 139.54, 137.20, 134.71, 130.62, 130.58, 123.65, 123.53, 118.35, 118.31, 113.26, 113.23, 113.20, 113.02, 112.99, 112.96, 110.95, 110.69, 109.44, 107.57, 107.51, 107.34, 107.27, 107.23, 107.02, 106.98, 106.49, 105.12, 98.97, 56.04, 55.67.

**M-2-2**

HRMS (ESI) m/z Calculated for $C_{35}H_{17}O_7F_{11}$:
$[M+H]^+$ theoretical mass: 759.08714, found 759.08682, difference -0.42 ppm.

$^1H$ NMR (400 MHz, $CDCl_3$) δ 8.05 (d, $J$ = 8.8 Hz, 1H), 7.17 (d, $J_{HF}$ = 10.4 Hz, 2H), 7.11 – 6.91 (m, 6H), 6.58 (d, $J$ = 8.8 Hz, 1H), 6.54 (s, 1H), 3.95 (s, 3H), 3.91 (s, 3H).

$^{19}F$ NMR (376 MHz, $CDCl_3$) δ -62.00 (t, 2F, $J$ = 26.5 Hz), -106.60 (d, 2F, $J_{HF}$ = 9.6 Hz), -110.58 (td, 2F, $J$ = 26.5, $J_{HF}$ = 10.4 Hz), -111.76 (d, 2F, $J_{HF}$ = $J$ = 8.5 Hz), -132.46 (dd, 2F, $J$ = 20.8, $J_{HF}$ = 8.0 Hz), -163.09 (tt, 1F, $J$ = 20.8, 5.7 Hz).

$^{13}C$ NMR (101 MHz, $CDCl_3$) δ 165.77, 163.05, 162.97, 162.76, 161.76, 161.02, 160.95, 160.87, 160.47, 160.39, 158.52, 158.49, 158.45, 158.39, 158.37, 155.78, 155.64, 155.49, 152.31, 152.26, 152.20, 152.15, 151.41, 151.27, 151.12, 149.81, 149.76, 149.70, 149.65, 144.59, 144.51, 144.40, 139.87, 139.71, 139.58, 139.56, 137.38, 137.22, 137.07, 134.71, 134.36, 134.25, 134.14, 122.62, 119.99, 117.35, 114.87, 114.84, 114.62, 114.60, 113.46, 113.31, 113.29, 113.10, 109.68, 109.37, 107.62, 107.55, 107.45, 107.38, 107.33, 107.29, 107.07, 107.03, 106.84, 106.82, 106.76, 106.63, 106.57, 106.54, 106.21, 106.05, 105.89, 105.13, 98.96, 56.03, 55.66.

## NMR Spectra

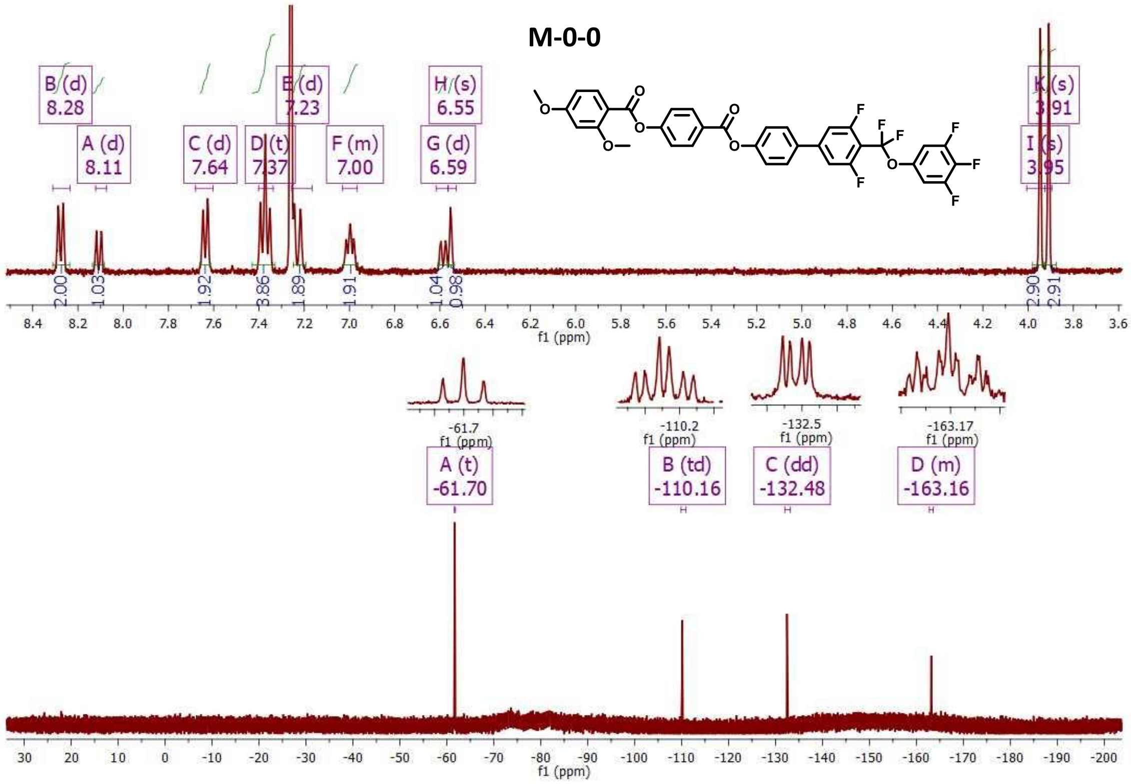


*Figure S1. $^{1}$H and $^{19}$F NMR spectra of **M-0-0**.*

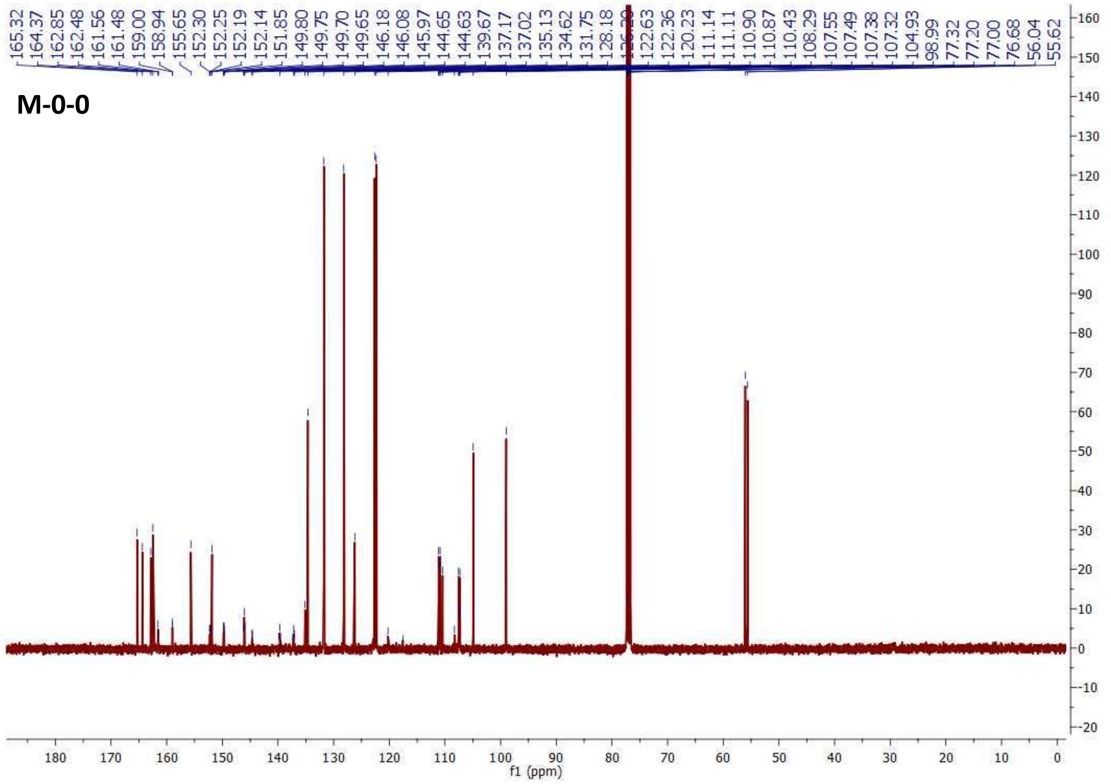


*Figure S2. $^{13}$C NMR spectrum of **M-0-0***

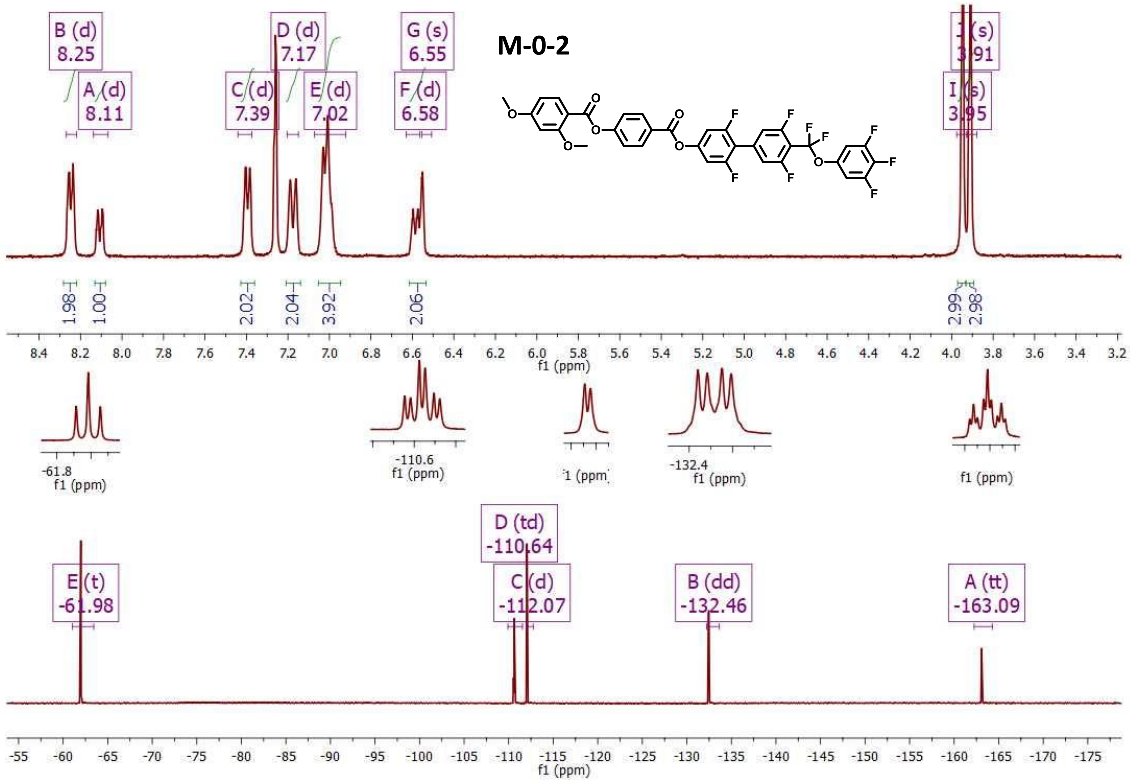


*Figure S3. $^{1}H$ and $^{19}F$ NMR spectra of **M-0-2**.*

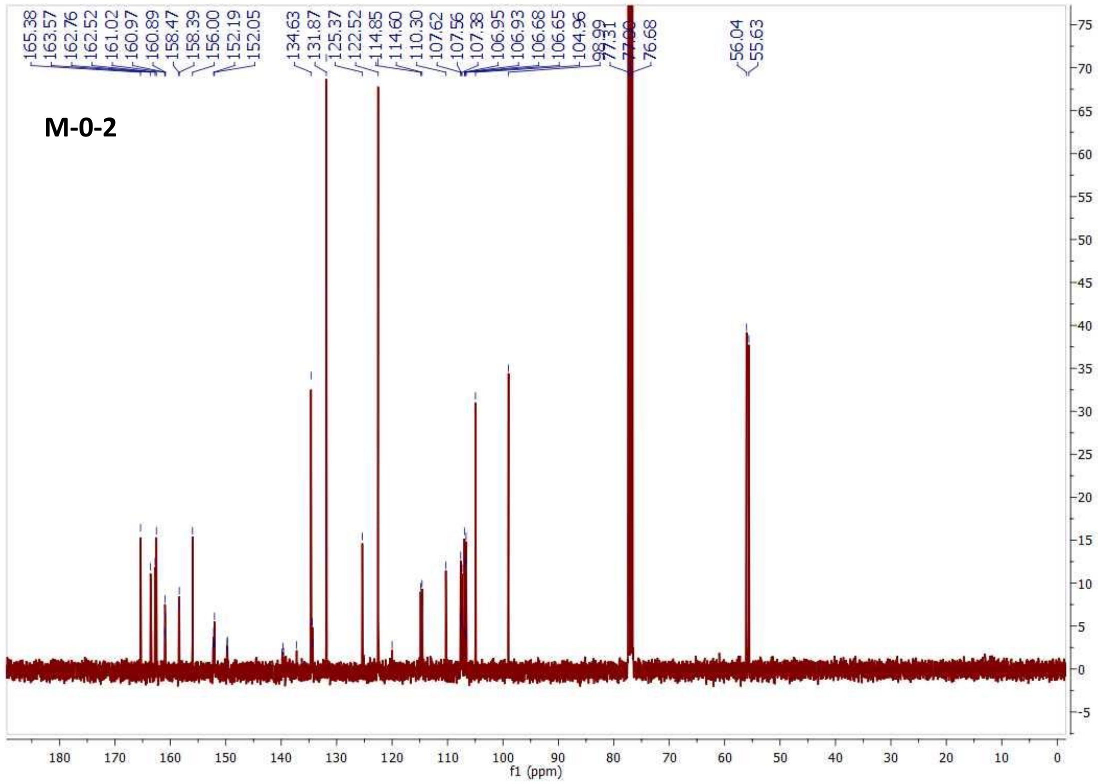


*Figure S4. $^{13}C$ NMR spectrum of **M-0-2**.*

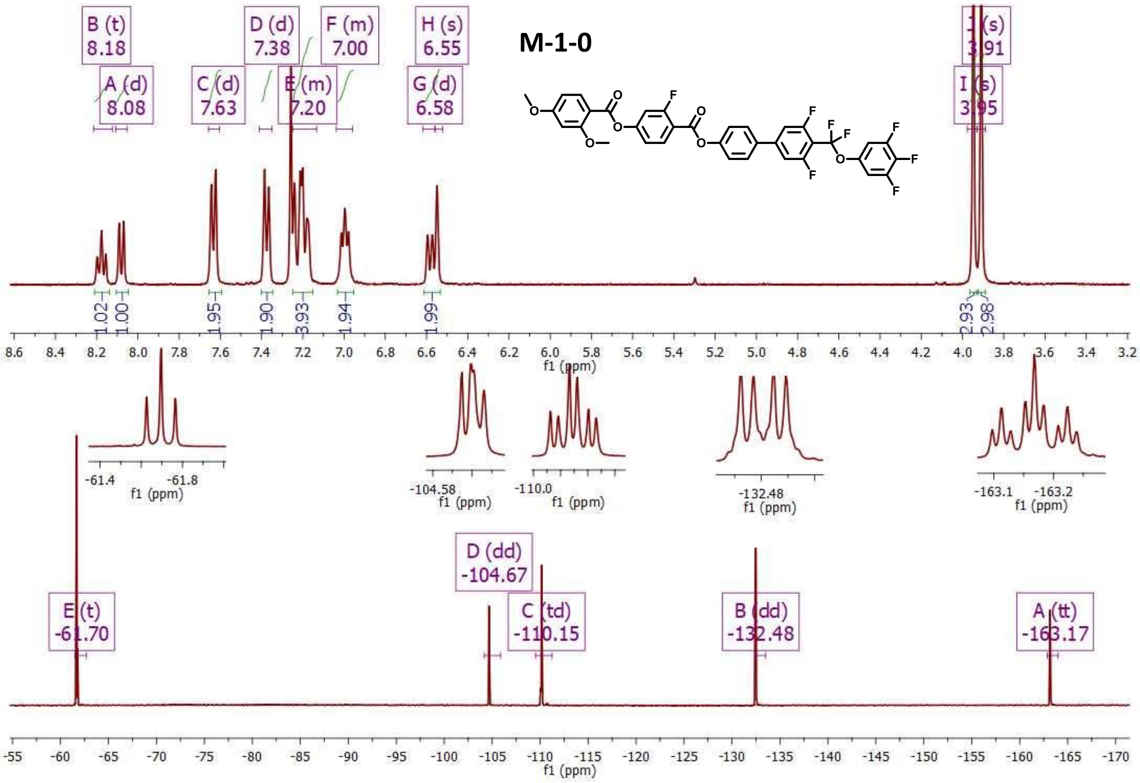


*Figure S5. $^1H$ and $^{19}F$ NMR spectra of **M-1-0**. The peak at 5.3 ppm in the $^1H$ spectrum is residual dichloromethane.*

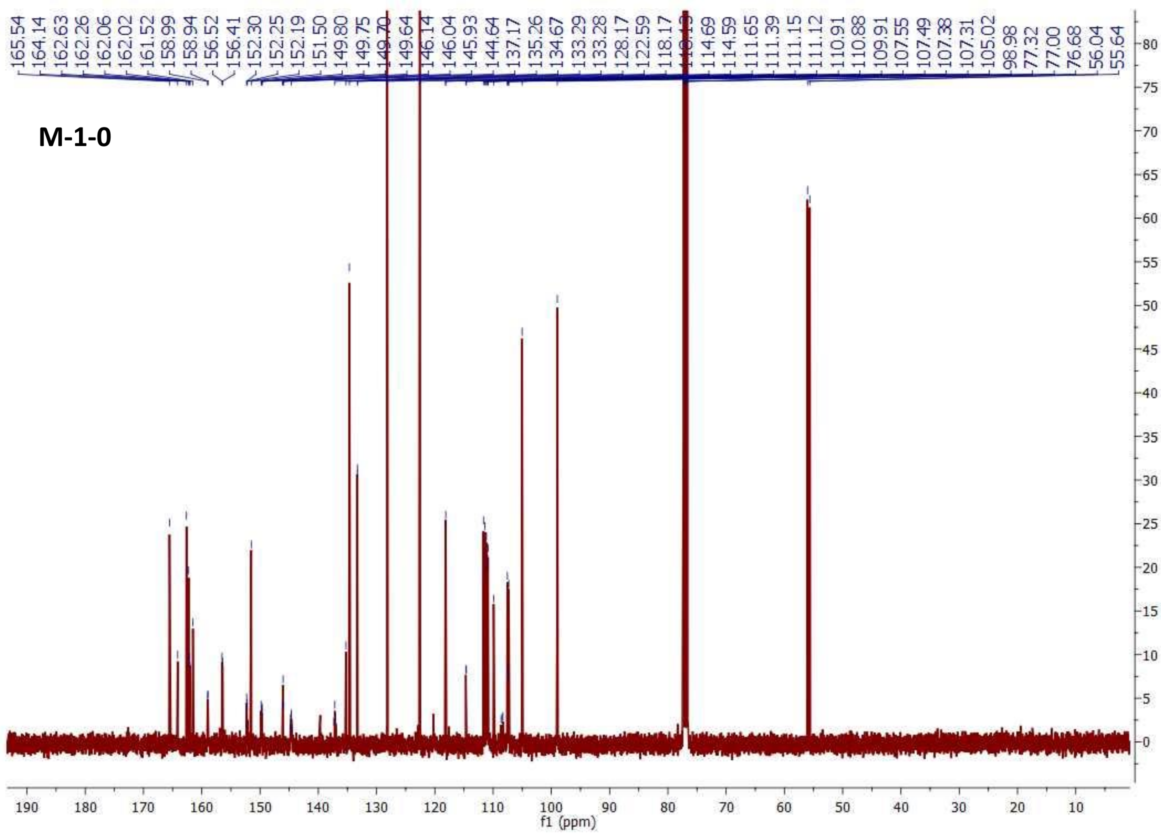


*Figure S6. $^{13}C$ NMR spectrum of **M-1-0**.*

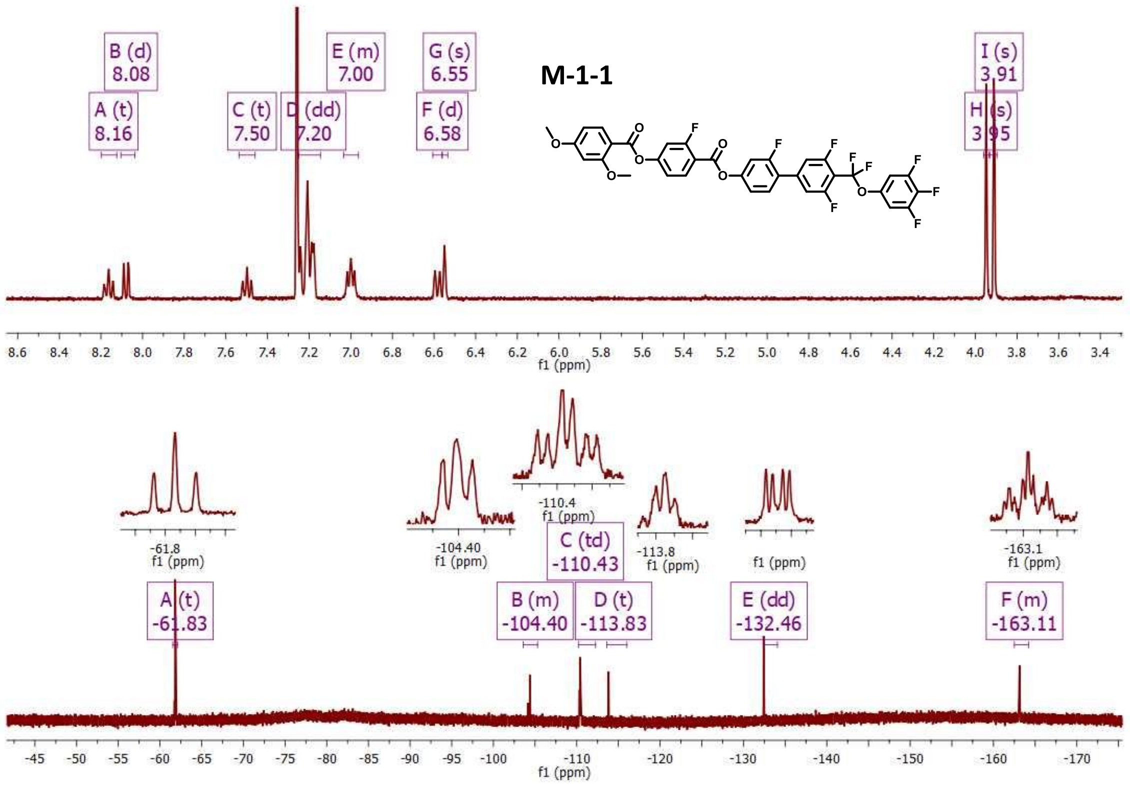


*Figure S7. $^{1}$H and $^{19}$F NMR spectra of **M-1-1**.*

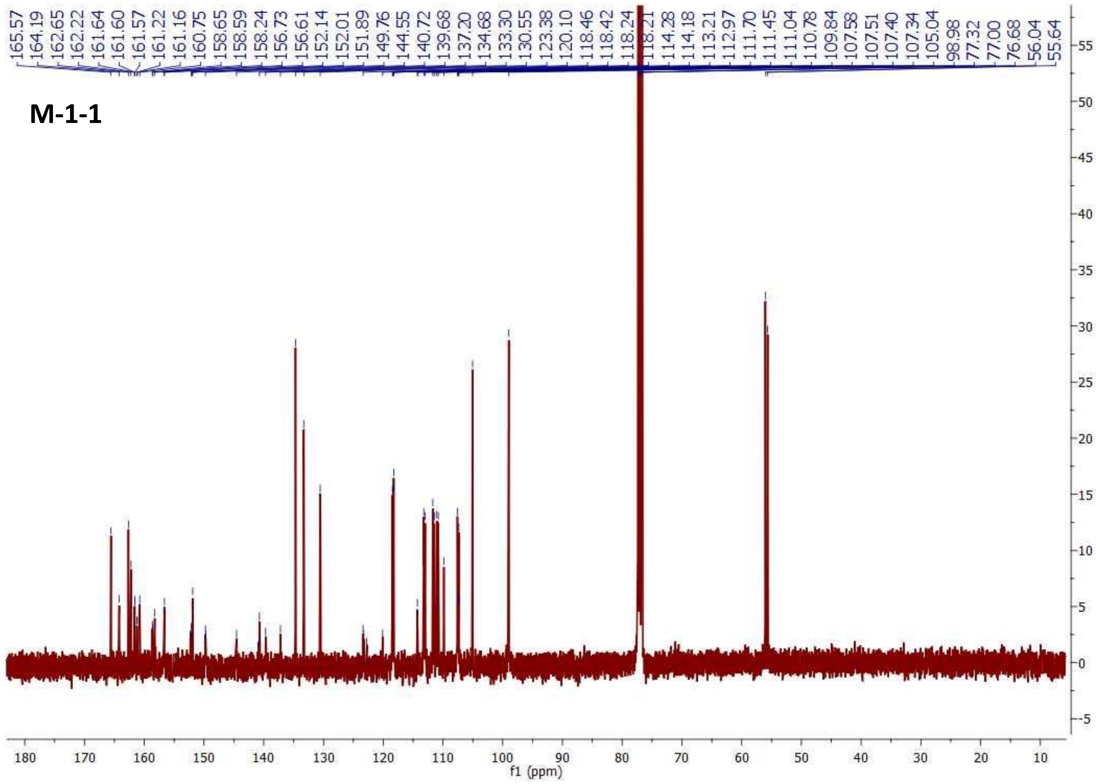


*Figure S8.$^{13}$C NMR spectrum of **M-1-1**.*

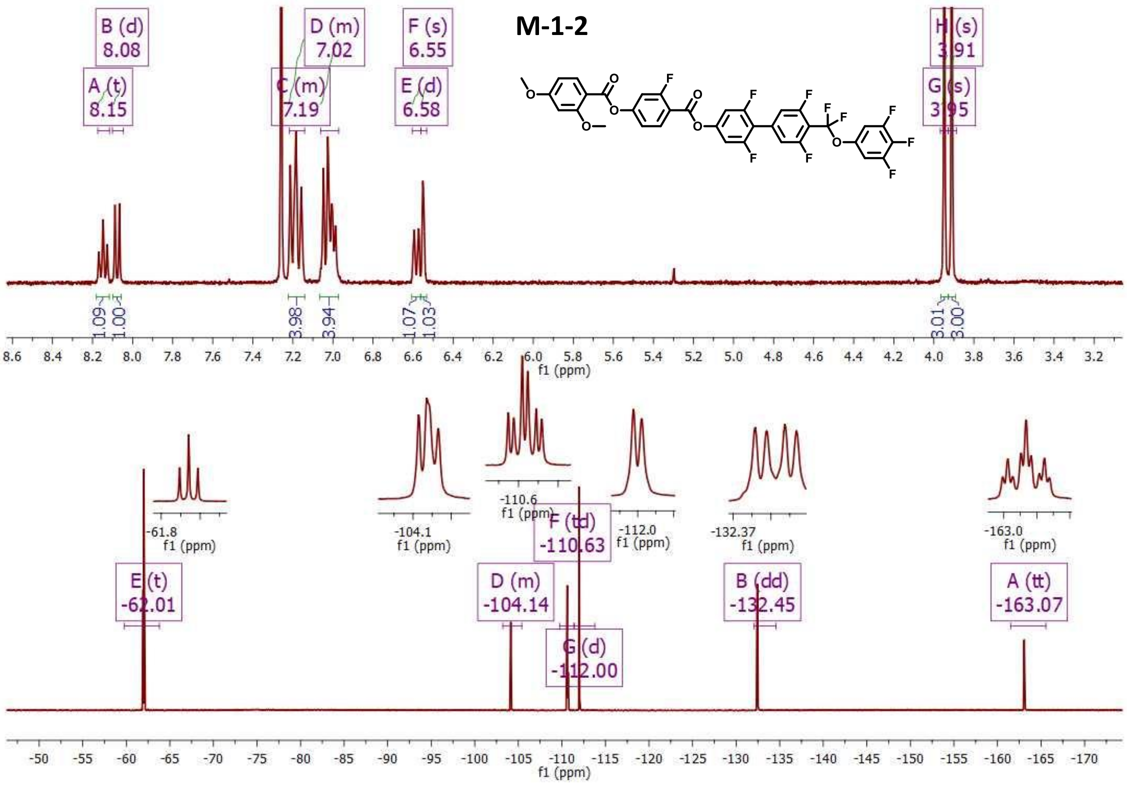


*Figure S9. $^{1}H$ and $^{19}F$ NMR spectra of **M-1-2**. The peak at 5.3 ppm in the $^{1}H$ spectrum is residual dichloromethane.*

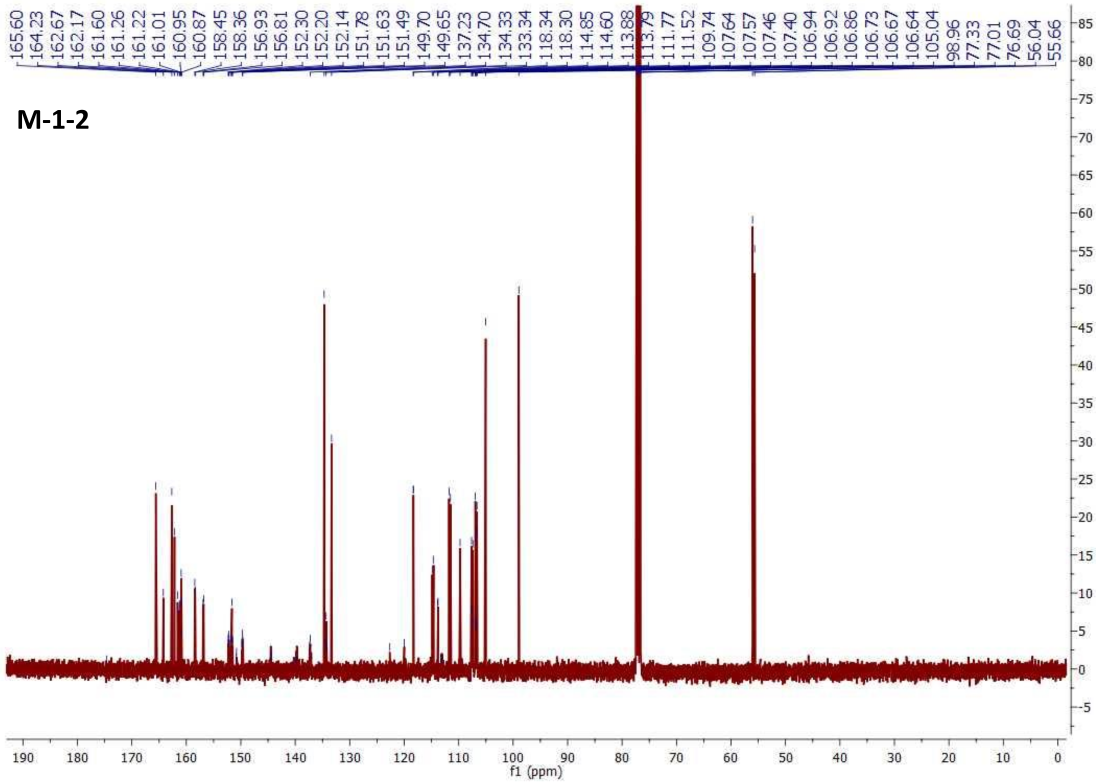


*Figure S10. $^{13}C$ NMR spectrum of **M-1-2.***

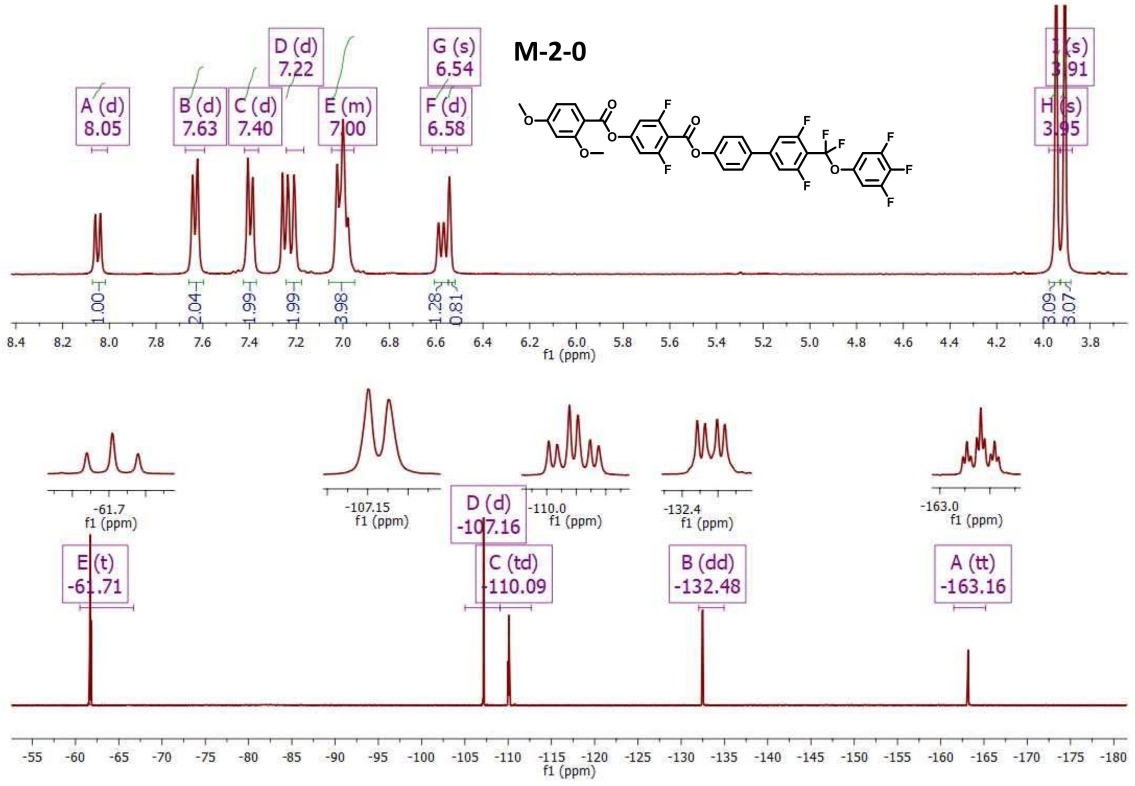


*Figure S11. $^{1}H$ and $^{19}F$ NMR spectra of **M-2-0**.*

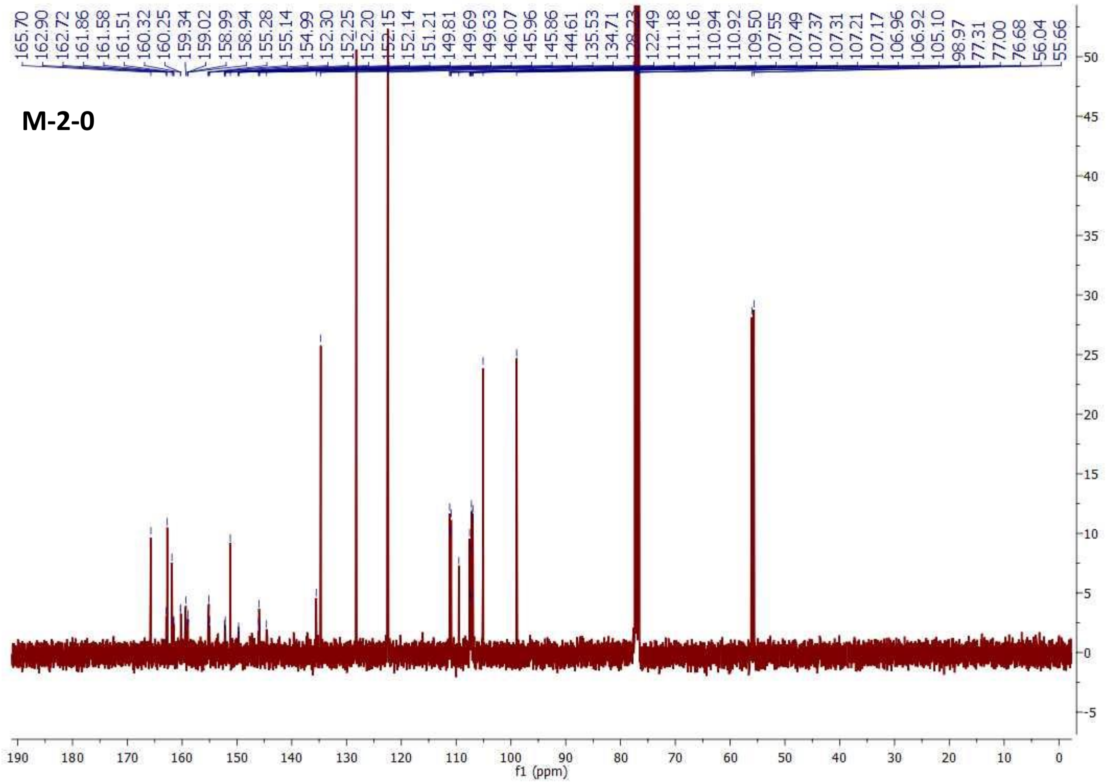


*Figure S12. $^{13}C$ NMR spectrum of **M-2-0**.*

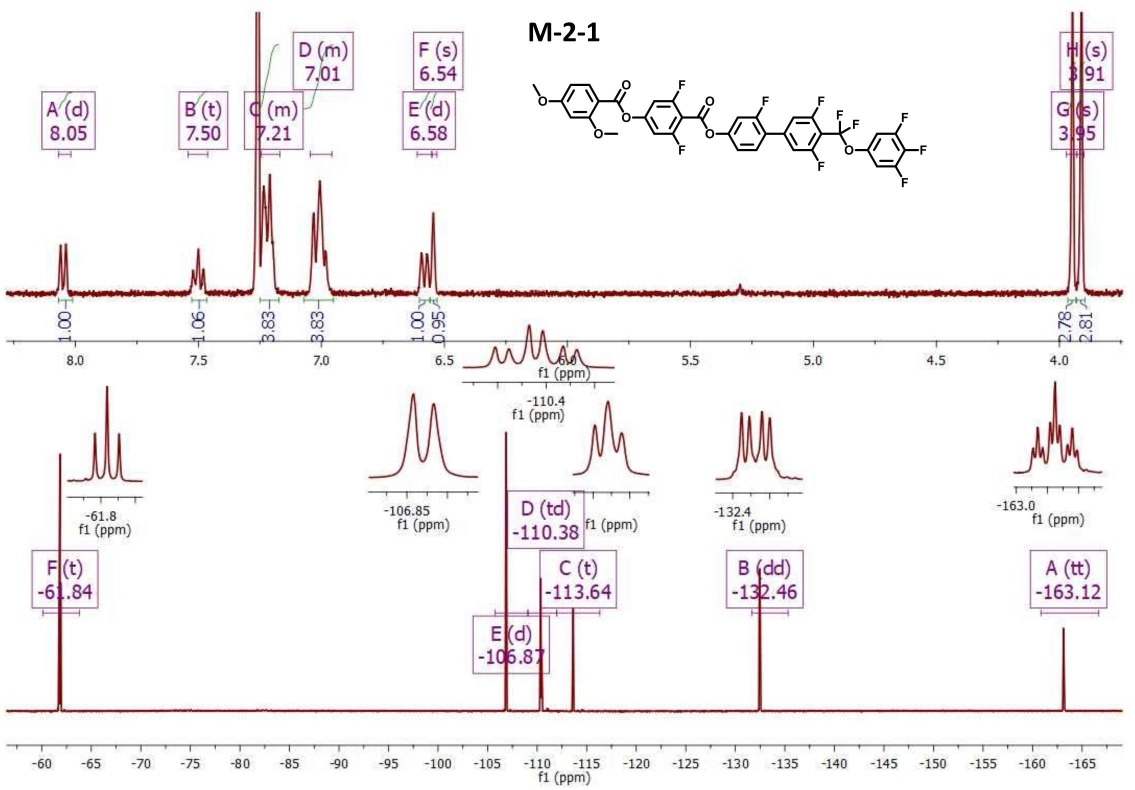


*Figure S13. $^{1}H$ and $^{19}F$ NMR spectra of **M-2-1**. The peak at 5.3 ppm in the $^{1}H$ spectrum is residual dichloromethane.*

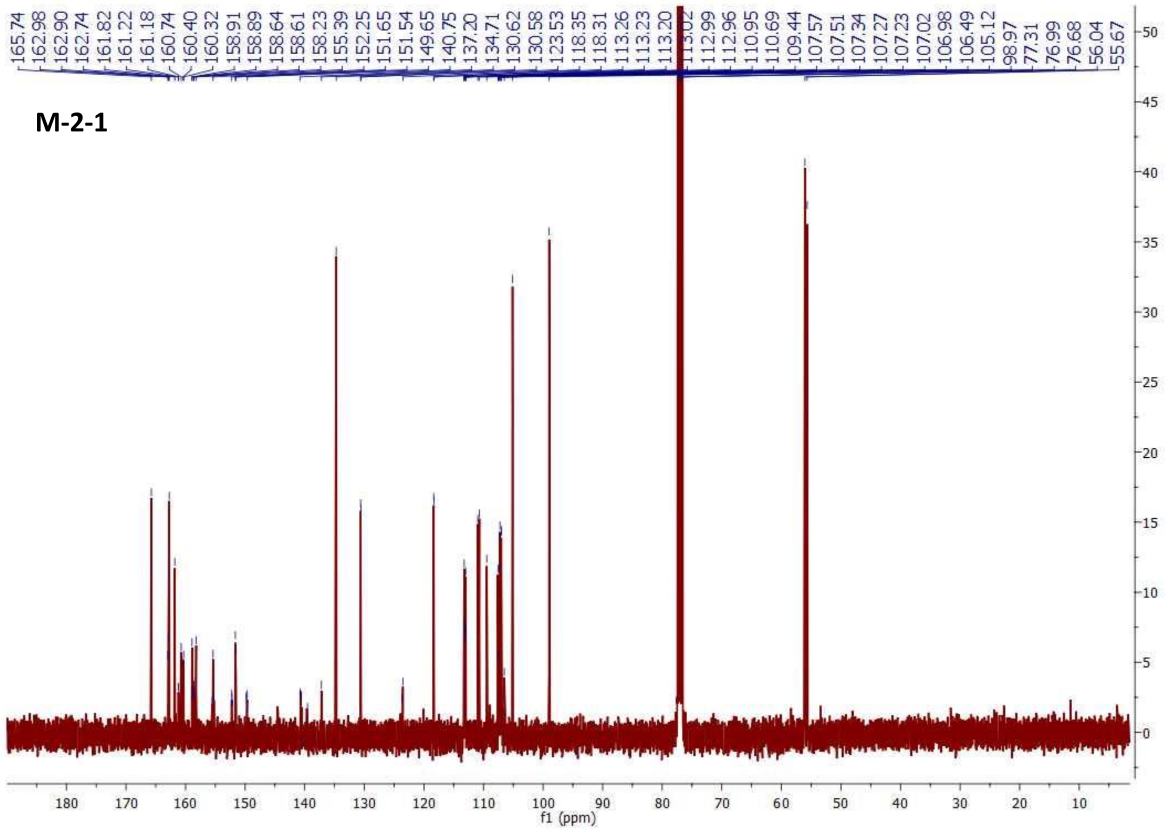


*Figure S14. $^{13}C$ NMR spectrum of **M-2-1**.*

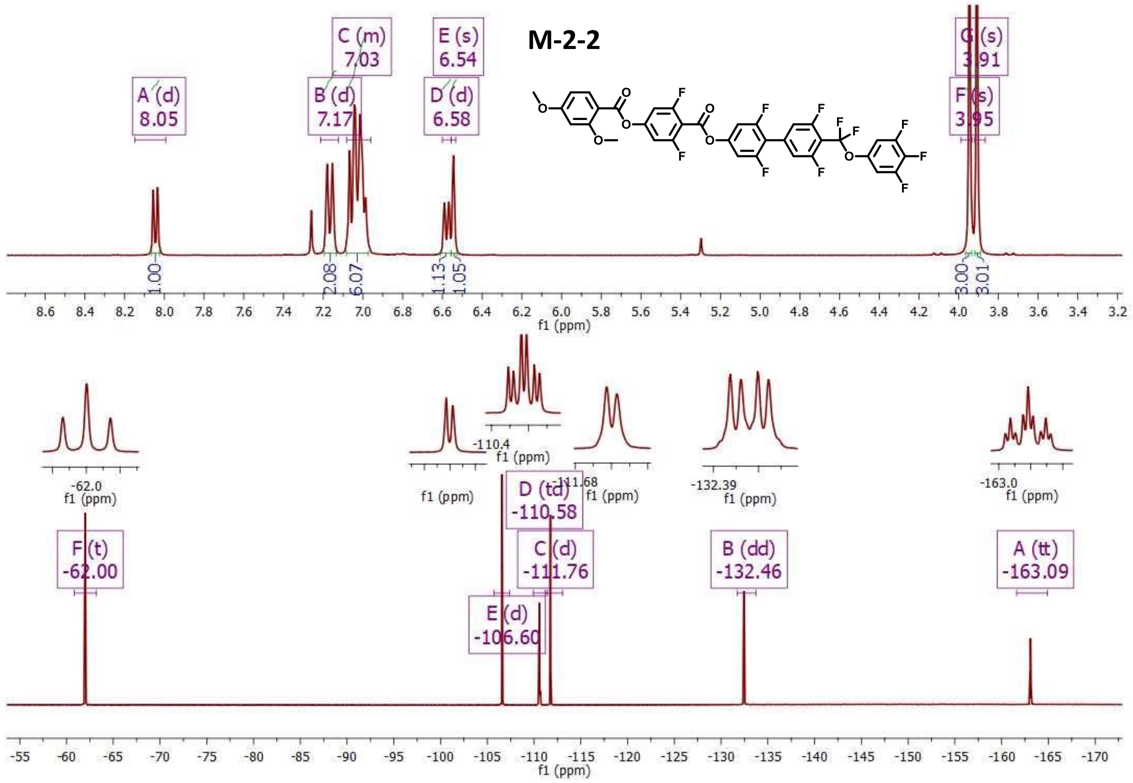


*Figure S15. $^{1}H$ and $^{19}F$ NMR spectra of **M-2-2**. The peak at 5.3 ppm in the $^{1}H$ spectrum is residual dichloromethane.*

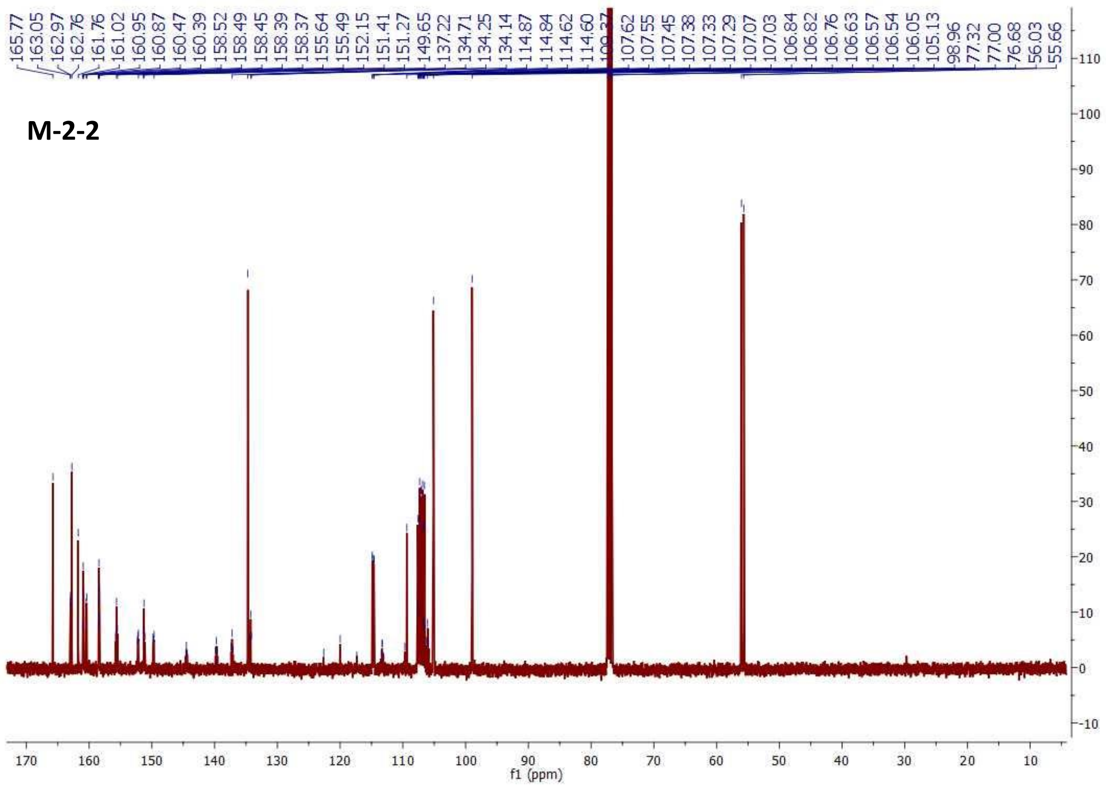


*Figure S16. $^{13}C$ NMR spectrum of **M-2-2**.*

## Additional results related to structure of observed mesophases

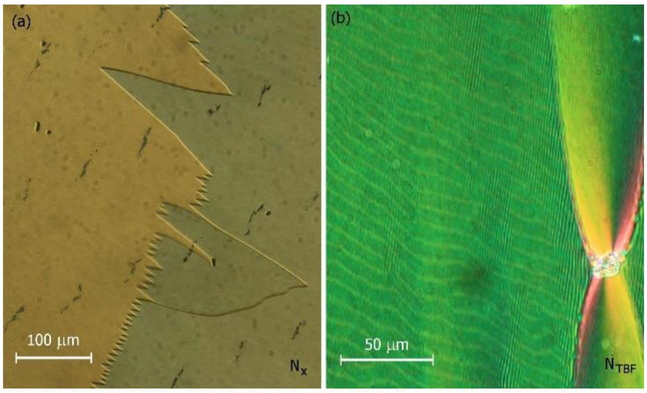


*Figure S17. Optical textures: (a) chevron texture characteristic for $N_X$ phase, observed for compound* ***M-1-0*** *in a 1.6-µm-thick cell with planar anchoring condition and (b) striped texture observed for $N_{TBF}$ phase of compound* ***M-1-2*** *in 5-µm-thick cell with planar anchoring. The periodicity of thin lines visible in (b) is related to the helical pitch.*

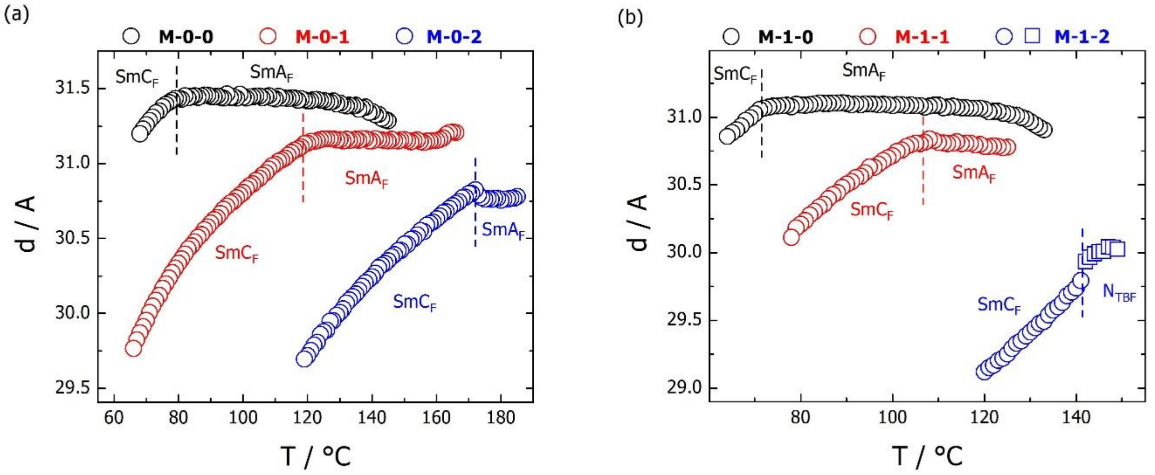


*Figure S18. Smectic layer thickness vs. temperature for (a)* ***M-0-Y*** *and (b)* ***M-1-Y*** *compounds. Transition to tilted $SmC_F$ phase is clearly visible as a decrease of layer spacing. Note, that for* ***M-1-2*** *a $N_{TBF}$ phase is formed above $SmC_F$ phase instead of the $SmA_F$ phase which is observed for other mesogens.*

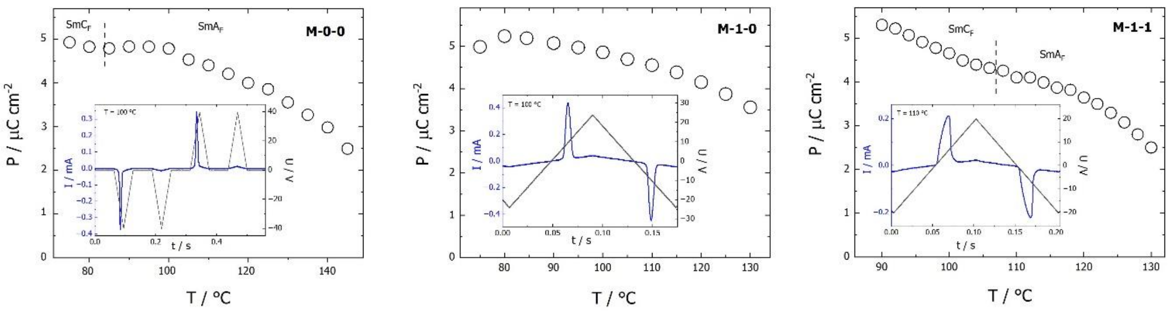


*Figure S19. Electric polarization, P, measured vs. temperature for chosen compounds. In the insets - switching current recorded under triangular- or modified triangular-wave voltage in $SmA_F$ phase. In modified triangular-wave scheme two consecutive pulses of the same polarity are applied; the first pulse gives the current peak while no current peak is observed with the second pulse, which confirms the ferroelectric ground state of the phase.*

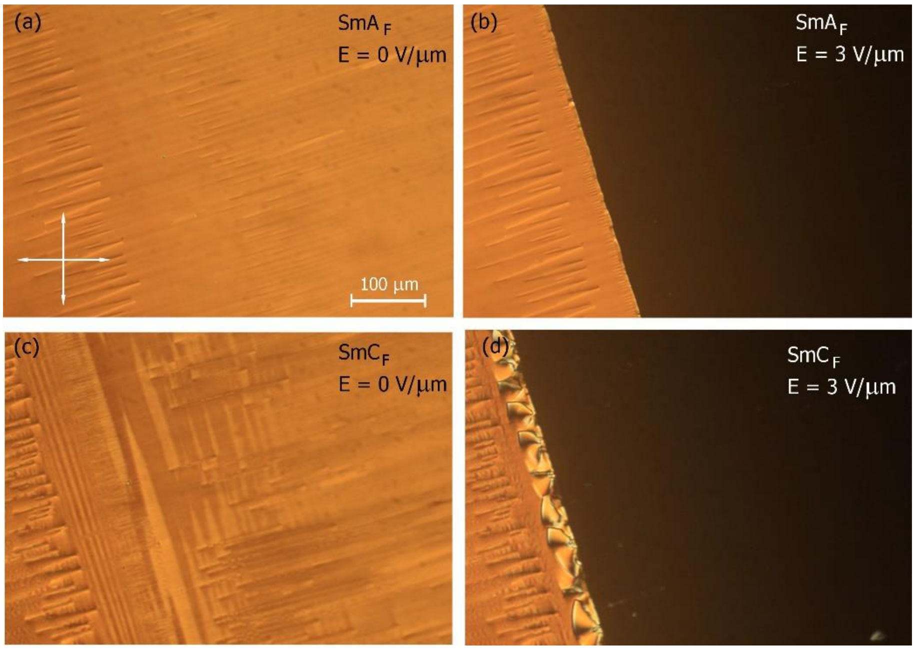


*Figure S20. The textures of (a,b) SmA$_F$ and (c,d) SmC$_F$ phases of **M-0-0,** observed in 1.8-μm-thick cell with planar anchoring condition: (a,c) before and (b,d) under application of electric field across cell thickness. The perfect homeotropic alignment is obtained in the electrode area (right side of images) as molecules align along the applied electric field.*

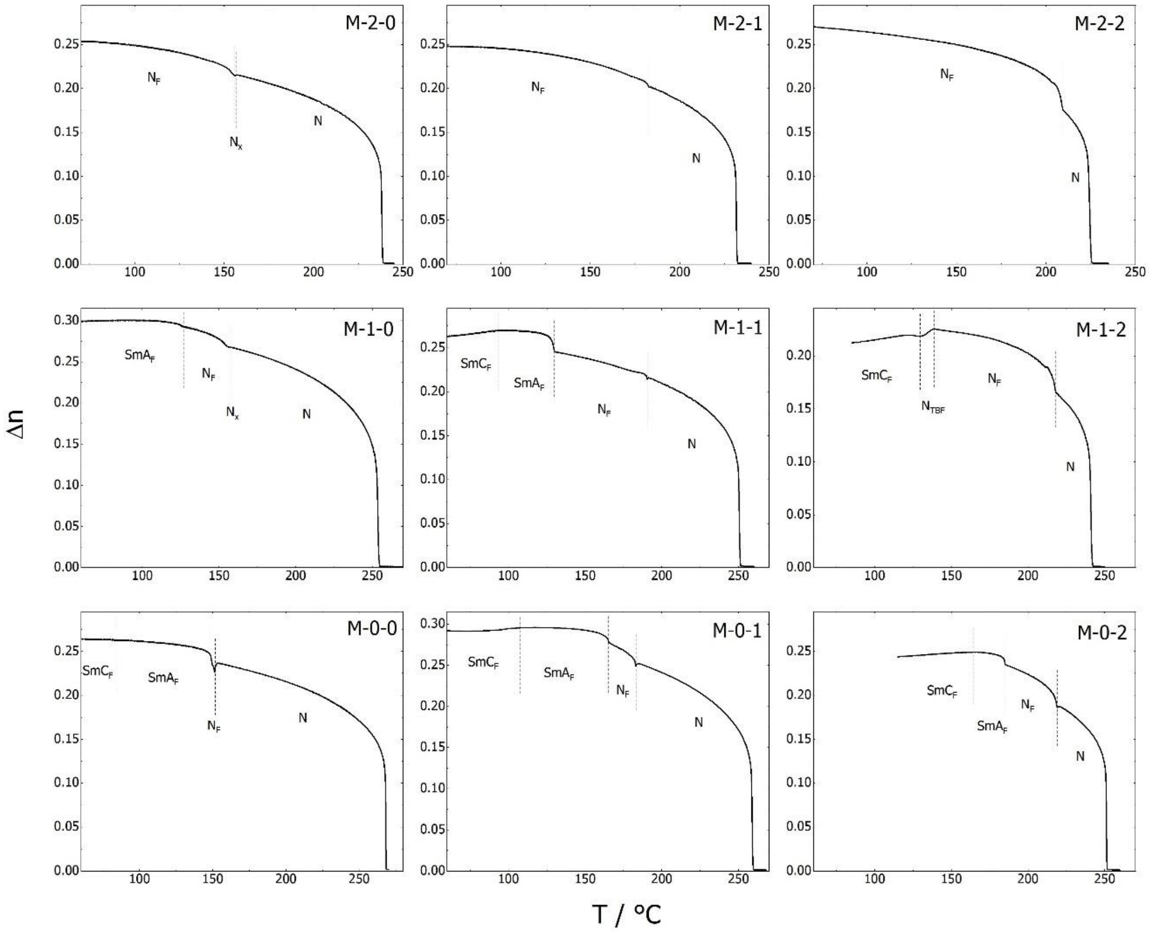


*Figure S21. Optical birefringence measured with green light ($\lambda$=532 nm) for **M-X-Y** compounds.*

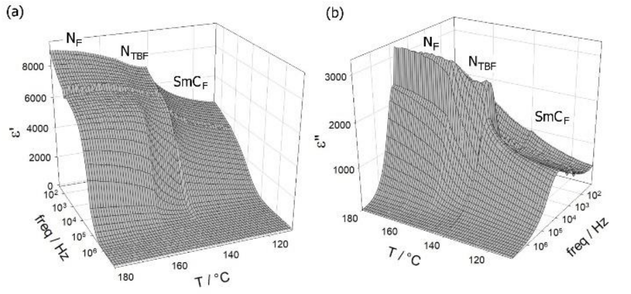


*Figure S22. Real (a) and imaginary (b) parts of apparent dielectric permittivity of **M-1-2** measured in a 5-μm-thick cell with gold electrodes and no surfactant layers.*

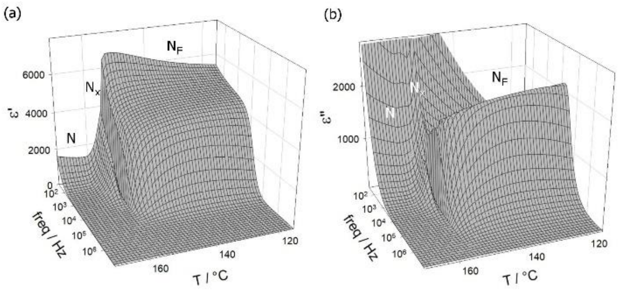

*Figure S23. Real (a) and imaginary (b) parts of apparent dielectric permittivity of **M-2-0** measured in a 5-µm-thick cell with gold electrodes and no surfactant layers.*

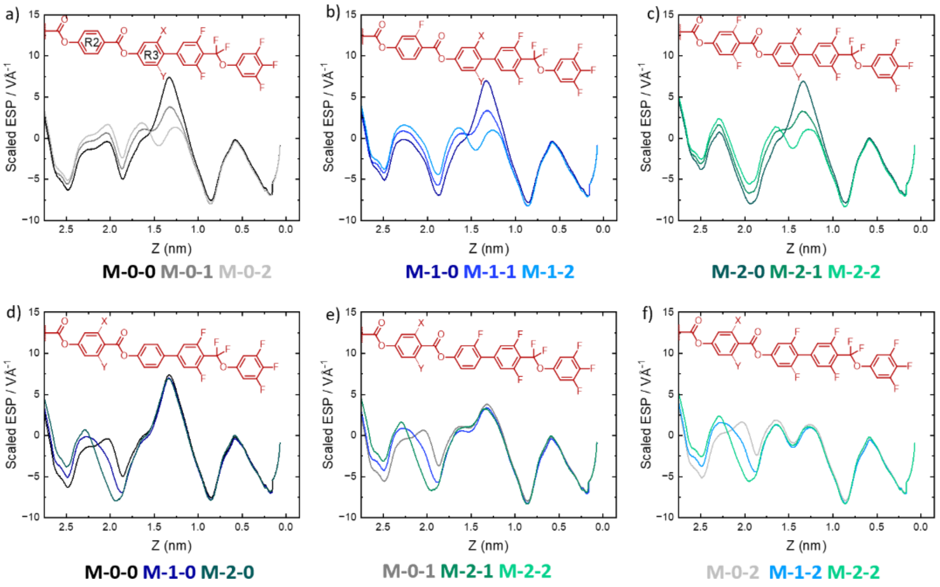

*Figure S24. 1D representations of the electrostatic potential along the long axis of the molecules comparing the effect of fluorination on ring 3 (a-c) and ring 2 (d-f).*

## References

(1) Frisch, M. J.; Trucks, G. W.; Schlegel, H. B.; Scuseria, G. E.; Robb, M. A.; Cheeseman, J. R.; Scalmani, G.; Barone, V.; Petersson, G. A.; Nakatsuji, H.; Li, X.; Caricato, M.; Marenich, A. V.; Bloino, J.; Janesko, B. G.; Gomperts, R.; Mennucci, B.; Hratchian, H. P.; Ortiz, J. V.; Izmaylov, A. F.; Sonnenberg, J. L.; Williams; Ding, F.; Lipparini, F.; Egidi, F.; Goings, J.; Peng, B.; Petrone, A.; Henderson, T.; Ranasinghe, D.; Zakrzewski, V. G.; Gao, J.; Rega, N.; Zheng, G.; Liang, W.; Hada, M.; Ehara, M.; Toyota, K.; Fukuda, R.; Hasegawa, J.; Ishida, M.; Nakajima, T.; Honda, Y.; Kitao, O.; Nakai, H.; Vreven, T.; Throssell, K.; Montgomery Jr., J. A.; Peralta, J. E.; Ogliaro, F.; Bearpark, M. J.; Heyd, J. J.; Brothers, E. N.; Kudin, K. N.; Staroverov, V. N.; Keith, T. A.; Kobayashi, R.; Normand, J.; Raghavachari, K.; Rendell, A. P.; Burant, J. C.; Iyengar, S. S.; Tomasi, J.; Cossi, M.; Millam, J. M.; Klene, M.; Adamo, C.; Cammi, R.; Ochterski, J. W.; Martin, R. L.; Morokuma, K.; Farkas, O.; Foresman, J. B.; Fox, D. J. Gaussian 16 Rev. C.01, 2016.

(2) Hobbs, J.; Gibb, C. J.; Mandle, R. J. Emergent Antiferroelectric Ordering and the Coupling of Liquid Crystalline and Polar Order. *Small Sci.* **2024**, *4*, 2400189. https://doi.org/10.1002/smsc.202400189.
(3) Gibb, C. J.; Hobbs, J.; Mandle, R. J. Systematic Fluorination Is a Powerful Design Strategy Towards Fluid Molecular Ferroelectrics. arXiv November 21, 2024. https://doi.org/10.48550/arXiv.2411.14115.
(4) Strachan, G. J.; Górecka, E.; Szydłowska, J.; Makal, A.; Pociecha, D. Nematic and Smectic Phases with Proper Ferroelectric Order. arXiv October 15, 2024. https://doi.org/10.48550/arXiv.2408.07381.
